# Microtubules provide force to promote membrane uncoating in vacuolar escape for a cyto-invasive bacterial pathogen

**DOI:** 10.1038/s41467-024-45182-6

**Published:** 2024-02-05

**Authors:** Yuen-Yan Chang, Camila Valenzuela, Arthur Lensen, Noelia Lopez-Montero, Saima Sidik, John Salogiannis, Jost Enninga, John Rohde

**Affiliations:** 1 Dynamics of Host-Pathogen Interactions Unit, Institut Pasteur, and CNRS UMR 3691 Université de Paris Cité, Paris, France; 2 Division of Molecular and Cellular Biology, Eunice Kennedy Shriver National Institute of Child Health and Human Development, National Institutes of Health, Bethesda, MD USA; 3 Department of Microbiology and Immunology, Dalhousie University, Halifax, NS Canada; 4 Department of Cellular and Molecular Medicine, University of California San Diego, La Jolla, CA USA; 5 Howard Hughes Medical Institute, Chevy Chase, MD USA; 6 Present Address: Department of Molecular Physiology and Biophysics, University of Vermont, Burlington, USA

**Keywords:** Pathogens, Small GTPases, Cellular microbiology, Biochemistry

## Abstract

Intracellular bacterial pathogens gain entry to mammalian cells inside a vacuole derived from the host membrane. Some of them escape the bacteria-containing vacuole (BCV) and colonize the cytosol. Bacteria replicating within BCVs coopt the microtubule network to position it within infected cells, whereas the role of microtubules for cyto-invasive pathogens remains obscure. Here, we show that the microtubule motor cytoplasmic dynein-1 and specific activating adaptors are hijacked by the enterobacterium *Shigella flexneri*. These host proteins were found on infection-associated macropinosomes (IAMs) formed during *Shigella* internalization. We identified Rab8 and Rab13 as mediators of dynein recruitment and discovered that the *Shigella* effector protein IpaH7.8 promotes Rab13 retention on moving BCV membrane remnants, thereby facilitating membrane uncoating of the *Shigella*-containing vacuole. Moreover, the efficient unpeeling of BCV remnants contributes to a successful intercellular spread. Taken together, our work demonstrates how a bacterial pathogen subverts the intracellular transport machinery to secure a cytosolic niche.

## Abstract (web summary)

*Shigella* enters human cells in a phagocytic vacuole and then escapes the vacuole to colonize the cytosol. Here, Chang and coworkers show that *Shigella* uses a bacterial effector to subvert host Rab proteins, microtubules and molecular motors to provide mechanical force to facilitate *Shigella* escape.

## Introduction

Eukaryotic cells possess innate immune defence mechanisms, which elicit pro-inflammatory responses or programmed cell death to eliminate pathogens^1^. Pathogenic microbes that invade eukaryotic hosts have developed strategies to avoid eliciting these innate immune programs^2^. Bacterial cell entry takes place within a bacteria-containing vacuole (BCV) that is derived from the plasma membrane of the infected cell. Some pathogens interrupt vacuole maturation and lysosomal degradation to propagate inside the BCV. On the other hand, cytosolic-dwelling bacteria rupture the BCV and replicate within the host cytosol, where they directly access nutrients^3^. An important strategy of cyto-invasive pathogens is avoidance of cellular detection, for example by the autophagy machinery recruited to damaged compartments or foreign particles. Consequently, the failure to uncoat BCV membranes potentiates the targeting of the microbes by host immune defence system.

Regardless of their replicative niches within host cells, many pathogenic microbes coopt the host cytoskeleton^4^. The cytoskeleton, consisting of microtubules, actin and intermediate filaments, is highly dynamic and is regulated by a repertoire of proteins in order to perform versatile functions including cell migration, maintaining cell shape, endocytosis and intracellular transport. Microtubules, for instance, are made up of protofilaments comprising heterodimers of α- and β-tubulins^5^. These protofilaments are generally nucleated from the centrosomal microtubule organization centre (MTOC) (also represents the microtubule minus-end), where the protofilaments grow to form the microtubule plus-end located generally near the cell periphery. For directional intracellular transport, the molecular motors cytoplasmic dynein-1 (dynein hereafter) and kinesins drive the delivery of vesicles and membranous organelles along polarized microtubule tracks^4,6^. Generally, plus-end-directed transport is executed by different kinesins, whereas minus-end-directed transport is primarily performed by dynein^7,8^. Dynein movement requires activation via complex formation with dynactin, where the dynein-dynactin interaction is strengthened by a family of coiled-coil-containing proteins termed activating adaptors^9^. Activating adaptors also serve to define the cargo specificity of dynein and can act as scaffolds to coordinate the movement of dynein and kinesin on the same cargo^10,11^.

Successful pathogenic bacteria and viruses exploit microtubules and motor proteins to remodel the cellular microenvironment for their intracellular niche establishment. One of the best-studied examples is *Chlamydia trachomatis* internalized within a vacuolar-like inclusion that uses secreted bacterial effector proteins to post-translationally modify tubulin and direct microtubules to spatially position the inclusion body within the infected cell^12–14^. Another intracellular pathogen *Salmonella* Typhimurium, which replicates within a *Salmonella*-containing vacuole (SCV), also takes advantage of specific bacterial effectors to interact with both dynein and kinesins for positioning its SCV at the juxtanuclear region, and for the formation of *Salmonella*-induced filaments for nutrition acquisition^15–17^. Several viruses similarly hijack host microtubules to position themselves close to the host nucleus. In the case of adenovirus, the virus reaches the nuclear pore by subverting dynein on the microtubule network. Kinesin motors are then recruited to supply opposing mechanic forces that shred the capsid to uncoat the viral genome^18,19^. Despite these reports, it is not known that other microbes exert multi-directional forces for membrane uncoating.

The enteroinvasive bacterial pathogen *Shigella flexneri* escapes the BCV efficiently within minutes after uptake into epithelial cells in a process where damaged BCV remnants are rapidly carried away from the pathogen. This contrasts with cytosolic *Salmonella* that remains in close contact with the broken SCV for minutes to hours^20–22^. It has been previously reported that the broken BCV membranes are instantly targeted by the host autophagy machinery^23^. We discovered that *Shigella* subverts infection-associated macropinosomes (IAMs) for the initial destabilization of the BCV^20^. Furthermore, unwrapping of BCV membranes from the intracellular pathogen involves Rab GTPases and the exocyst complex recruited on the IAMs^24^. Apart from that, *Shigella* has been proposed to evade targeting by the autophagy system via the action of the bacterial effector VirA which was thought to sever microtubules^25,26^. Later reports, however, showed that VirA possesses neither microtubule depolymerizing activity nor protease function^27,28^. Together, the role of microtubules in *Shigella* infection, and its escape from cytosolic host recognition has remained unclear.

Here, we report that the microtubule network is subverted by the pathogen to preferentially move the BCV membrane remnants away from the bacterium. We demonstrate that the motor protein dynein facilitates cytosolic release of the pathogen by actively pulling BCV membranes away from the intracellular bacteria. The efficient BCV disassembly and unpeeling is relevant to *Shigella* cell-to-cell spread and to avoid the canonical autophagy targeting. Furthermore, we reveal that these events require a cascade of BCV-IAM interactions, involving host factors, namely Rab13 as well as the bacterial effector IpaH7.8. The events leading to force generation rely on the E3 ubiquitin ligase activity of IpaH7.8. Together, we show that mechanical forces exerted by microtubule-based transport contribute to efficient membrane uncoating of *Shigella*.

## Results

### *Shigella* BCV membrane remnants dynamically move along microtubules

**Fig. 1:**
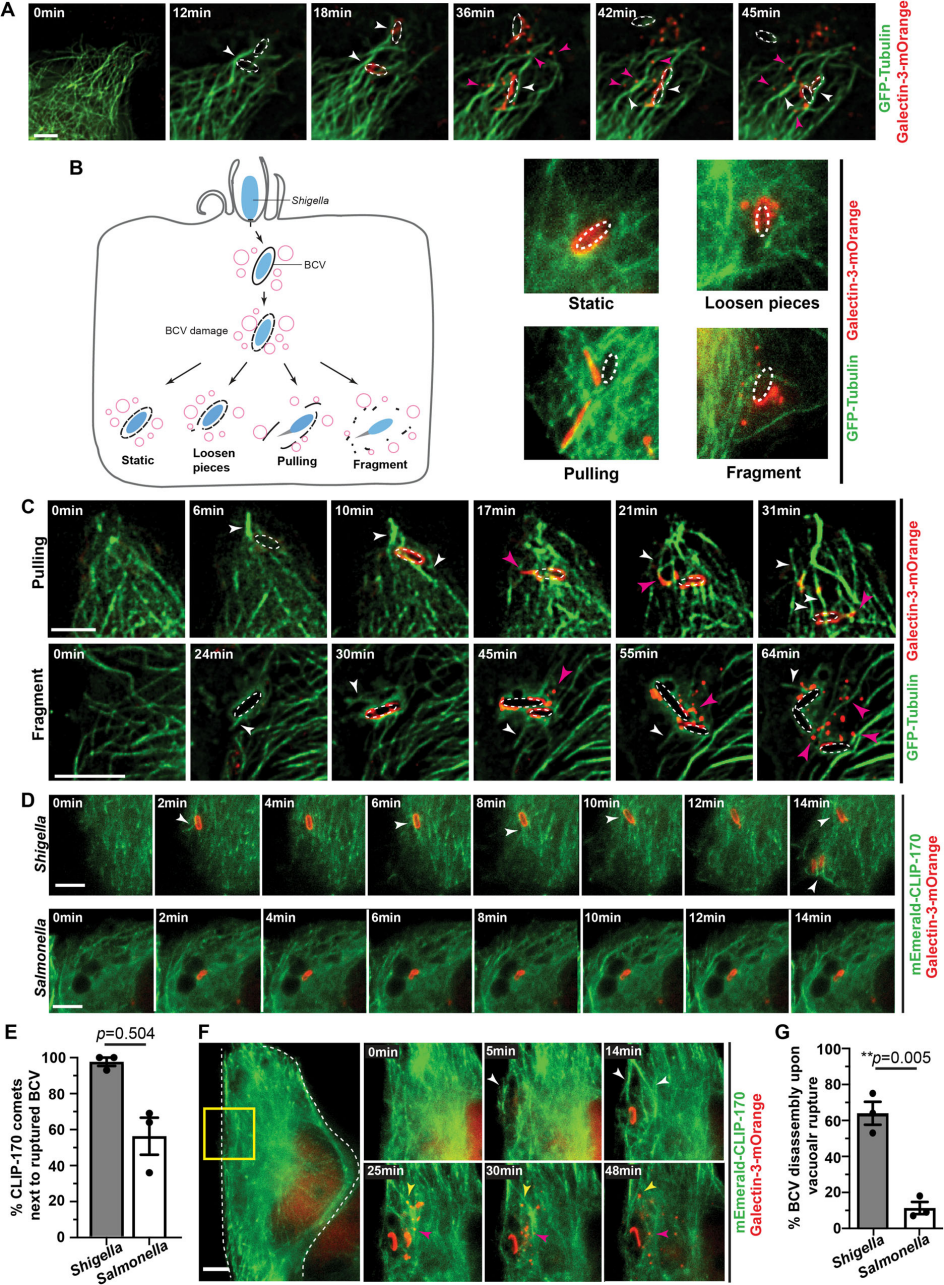
*Shigella* BCV membrane remnants move dynamically on microtubule networks. In all the time-lapse microscopic analysis, Galectin-3-mOrange was used as a marker for BCV damage and to trace the BCV membrane remnants, whereas all intracellular bacteria were indicated by white dotted ovals. Images were captured every min unless specified. Maximum z-projection of stacks of the representative infection foci are shown. Scale bars are 5 μm. **A** Analysis of *Shigella* BCV membrane remnants moving on microtubules after initial BCV damage. White arrowheads indicate the newly formed microtubules around the perforated BCV. Magenta arrowheads indicate the BCV membrane remnants moving on microtubules. **B** Graphical illustration of the different disassembly states of the perforated BCV upon initial vacuolar damage. Static: BCV did not disassemble; Loosen pieces: Small pieces of membrane moved away from the damaged BCV, while the outline of the vacuole remained; Pulling: Segments of BCV being pulled away from the bacteria; Fragment: damaged BCVs got fragmented and vanished. **C** Microscopic images of the pulling and fragment phenotype as illustrated in **B**. Images were captured every 30 s. White arrowheads indicate the microtubules formed in situ. Magenta arrowheads indicate the moving membranes on microtubules. **D** Analysis of CLIP-170 localization in *Shigella-* or *Salmonella-*infected cells. White arrowheads indicate the CLIP-170 comets around ruptured BCVs. **E** Quantification of CLIP-170 localization next to ruptured BCV in *Shigella* or *Salmonella* infection (as observed in **D**). **F** Analysis of CLIP-170 localization in *Shigella* infection. The region highlighted in yellow is presented. White arrowheads indicate the enriched CLIP-170 around ruptured BCVs. Yellow or magenta arrowheads indicate the moving membranes. **G** Analysis of BCV disassembly in *Shigella* or *Salmonella* infection upon vacuolar rupture. Data in Fig. 1 are represented by mean ± SEM of three independent replicates. Statistical analysis used two-tailed Welch’s t-test, with reported *p*-values for significance comparison (ns: non-significant, **p < 0.01). Source data are provided as a Source Data file.

We aimed to understand the interplay between vacuolar membrane uncoating of a bacterial pathogen and host microtubules. We monitored the successive events upon *Shigella* entry into a host cell, in particular the displacement of *Shigella* BCV membrane after initial BCV damage by the appearance of the vacuolar rupture marker galectin-3 on the damaged vacuoles^29^. Using fluorescently labeled *Shigella*, we noticed the polymerization of microtubules in situ at the entry foci prior to initial BCV damage, whereas the galectin-3 signals only appeared in close vicinity to the bacterium outlining the damaged BCV (Supplementary Fig. 1A). We also found that the majority (> 90%) of the BCV membrane remnants appeared to be actively carried away along microtubules, switching among different microtubule tracks (Fig. 1A). By tracing the vacuolar rupture marker galectin-3, we then classified the different disassembly states of individual BCVs. About 30% of BCVs did not disassemble throughout the acquired movies (Fig. 1B, Supplementary Fig. S1B, “Static” and supplementary movie 1). The remaining ~70% of BCVs underwent disassembly into three observable phenotypes (Fig. 1b): (i) 16% of BCVs had pieces that loosened from the damaged vacuoles and moved away from the damaged vacuoles, while the outline of the vacuole remained in the acquired movies (Fig. 1B, Supplementary Fig. 1B, “Loosen pieces” and Supplementary Movie 2); (ii) 26% of BCVs showed segments being pulled away from the bacteria (Fig. 1B, C, top panel, Supplementary Fig. 1B, “Pulling” and Supplementary Movie 3) and (iii) 19% of BCVs fragmented within 5–15 min after which the damaged BCVs vanished (Fig. 1B, C, bottom panel, Supplementary Fig. 1B, “Fragment” and Supplementary Movie 4). In some cases, the BCVs exhibited more than one of the above phenotypes during the course of the acquired movies.

We next tracked the fate of individual bacteria and the broken BCV remnants under the treatment of the microtubule-depolymerizing drug nocodazole and microtubule-stabilizing drug taxol by time-lapse microscopy as described previously^24^. Although statistically non-significant, the presence of either drug resulted in a clear trend in increased population of bacteria being entrapped in the perforated BCVs (Supplementary Fig. 1, “Entrapped”) and a decrease in the occurrence of *Shigella* moving free of any membrane remnants (Supplementary Fig. 1C, “Free”). By employing our previously reported quantitative analysis method^24^, we observed that the drug treatment resulted in a significant delay in the timing of BCV disassembly (Supplementary Fig. 1D) and in the timing of *Shigella* vacuolar escape (Supplementary Fig. 1E). These results indicate that the displacement of the membrane remnants is significantly impaired when the microtubule network is disrupted.

Since we observed in situ polymerization-depolymerization of microtubules at *Shigella* infection foci (Fig. 1A), we hypothesized that the membrane remnants are moving along newly formed microtubules. The constant shrinkage and growth of microtubules are modulated by microtubule-associated proteins and microtubule plus-end tracking proteins ( + TIPs)^30,31^. One of the +TIPs is cytoplasmic linker protein of 170 kDa (namely CLIP-170 or CLIP1), which regulates microtubule dynamics and links molecular cargoes to the microtubule tracks^32,33^. We found that condensed CLIP-170 “comets” were observed next to nearly all perforated *Shigella* BCVs (Fig. 1D, E, “*Shigella*”). CLIP-170 comets were in close proximity to the moving membranes, suggesting a possible role of CLIP-170 in modulating the dynamic movement of the membrane remnants (Fig. 1F). We wondered if the uniform CLIP-170 localization to BCVs was unique to *Shigella* infection, or whether it was common to other bacterial pathogens. Therefore, we monitored the infection of *Salmonella* Typhimurium, which initiates damage of *Salmonella*-containing vacuole within the first hour post-invasion^22^, and tested the localization of CLIP-170 in relation to vacuolar damage (Fig. 1D, “*Salmonella*”). Surprisingly, we observed that CLIP-170 comets were bordered to only about 55% of the damaged *Salmonella* vacuoles (Fig. 1S). Whereas the vast majority of *Shigella* BCV membrane remnants disassembled, we observed the inverse for the *Salmonella* vacuole where ~90% of the measured broken vacuoles remained closely attached to the bacteria during the measured time courses (Fig. 1G). Moreover, we rarely observed membrane pulling or fragmentation of *Salmonella* vacuoles but did occasionally observe broken pieces of the vacuole remnants in close proximity to the damaged vacuole (Supplementary Fig. 1B). These results suggest that the bacterial pathogen *Shigell*a uniquely hijacks the microtubule network for displacing its BCV membrane remnants to facilitate vacuolar membrane uncoating.

### Dynein plays a role in moving BCV membrane remnants during *Shigella* infection

**Fig. 2:**
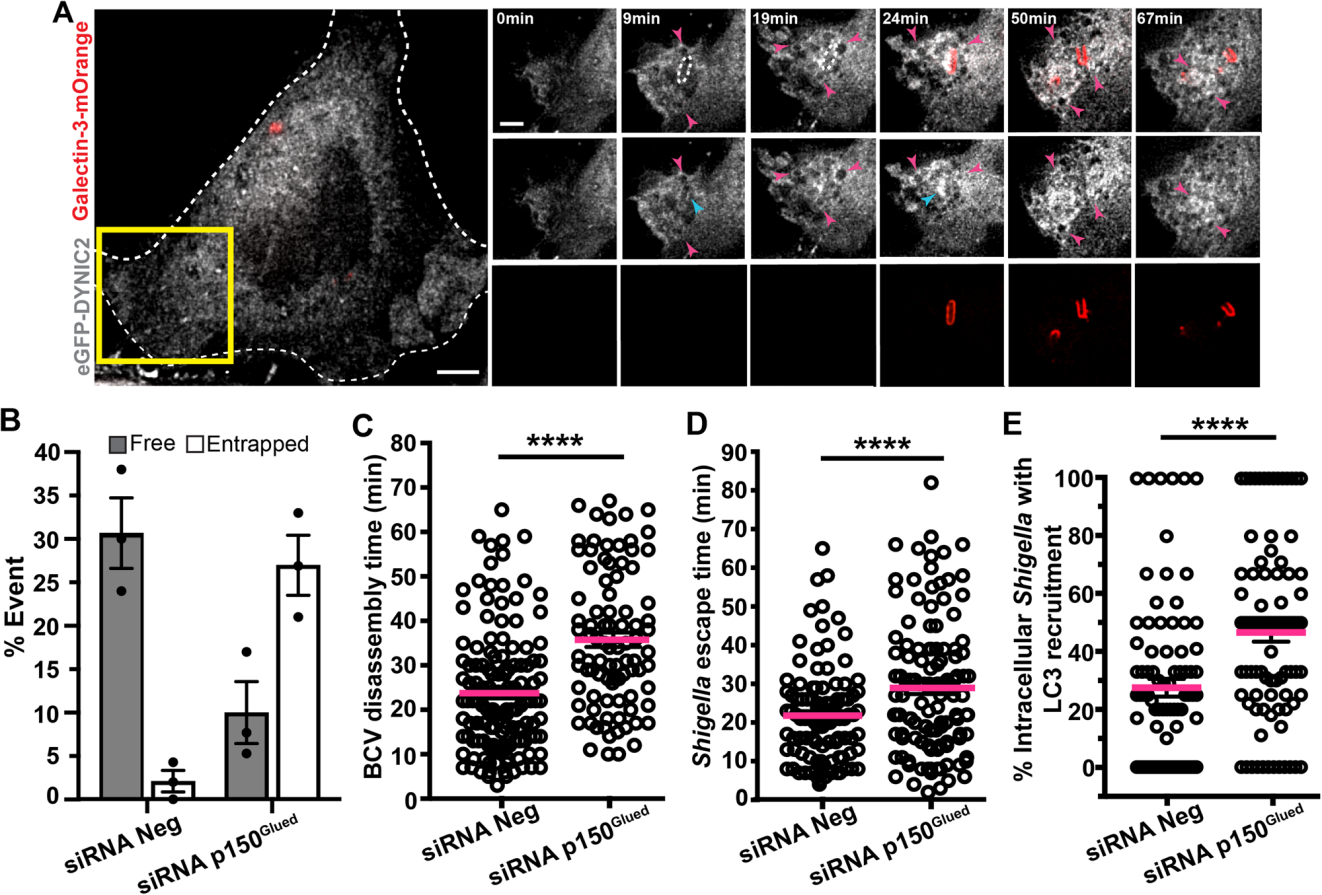
Dynein plays a role in moving BCV membrane remnants during *Shigella* infection. **A** Time-lapse microscopic images of the recruitment of dynein, as marked by DYNIC2 (gray) in transfected HeLa cells. Galectin-3-mOrange (red) was used as a marker of vacuolar rupture. Images were captured every min and maximum z-projection of stacks of the representative infection focus is shown. Blue arrowheads indicate the localization of dynein on BCV remnant while the magenta arrowheads indicate some dynein-positive infection-associated macropinosomes. Intracellular bacteria were indicated by white dotted ovals. Scale bars are 5 μm. **B** Analysis of the fates of individual *Shigella* in control HeLa cells and cells subjected to p150^Glued^ subunit depletion (siRNA p150^Glued^). The bars represent mean ± SEM of three independent replicates. Time-lapse microscopic analyses of *Shigella* infection of cells subjected to RNA interference of non-targeting control (siRNA Neg) versus p150^Glued^ subunit depletion (siRNA p150^Glued^) were performed to examine **C** time of *Shigella* BCV disassembly and **D** *Shigella* escape time (i.e. formation of actin tails). *n* > 90 infected cells in three independent replicates in each condition. The bars (magenta) represent the mean and unpaired t-tests were performed. **E** Analysis of the recruitment of LC3 to intracellular *Shigella* in infected cells at 45 min-post infection in cells subjected to RNA interference of non-targeting control (siRNA Neg) versus p150^Glued^ subunit depletion (siRNA p150^Glued^). *n* > 85 infected cells in three independent experiments in each condition. Data are represented by mean ± SEM of three independent replicates. Statistical analysis used two-tailed Welch’s t-test, with reported *p*-values for significance comparison (ns non-significant; ****p < 0.0001). Source data are provided as a Source Data file.

Dynein and kinesins drive long-range delivery of membranous organelles to their designated locations inside a cell^8^. Membrane remnants of the perforated BCVs were clearly displaced on microtubules during *Shigella* infection of epithelial cells, where switching between microtubule tracks were frequently observed. While analyzing the proteomes of IAMs recruited to the sites of vacuolar breakage, we identified the main constituents of the dynein complex being specifically enriched in the case of *Shigella*, but not for *Salmonella*^24,34^ (Supplementary Table 1). Therefore, we speculated that dynein may be involved in BCV remnant motility. To test this, we performed time-lapse microscopy during bacterial invasion in cells expressing a fluorescently labeled dynein complex (as marked by dynein intermediate chain 2, DYNIC2). DYNIC2 localized to BCV membrane remnants that were later being pulled away from *Shigella* (Fig. 2A and Supplementary Movie 5). We also confirmed the recruitment of endogenous dynein (as marked by DYNC1I1) on *Shigella* IAMs around the invading *Shigella* (Supplementary Fig. 2A). These data pinpointed a role of dynein in BCV unpeeling during *Shigella* entry.

To further investigate the role of the dynein motor complex, we perturbed its function by depleting p150^Glued^ subunit of dynactin (siRNA p150^Glued^) and examined the vacuolar escape of *Shigella*. We observed a negligible difference in the timing of the initial timepoint of BCV rupture (as marked by the appearance of gelectin-3 on BCV) in p150^Glued^ knock-down cells compared with the control (Supplementary Fig. 2B). However, we found a 3-fold decrease in the *Shigella* population without BCV membrane remnants attached at the onset of motility in p150^Glued^-depleted cells compared to control (Fig. 2B, “Free”). Cells depleted for p150^Glued^ resulted in more than 10-fold increase in *Shigella* confined in the perforated BCV ( < 2% in control and ~27% in p150^Glued^ depletion) (Fig. 2B, “Entrapped”). Furthermore, BCV disassembly was delayed in p150^Glued^-depleted cells (35.7 ± 1.6 min) when compared with the control (average 23 min) (Fig. 2C). Finally, *Shigella* took a longer time to acquire actin-based motility in p150^Glued^ depletion (28.9 ± 1.5 min) than control (21.8 ± 1.2 min) (Fig. 2D). An earlier report had implicated the ability of *Shigella* to evade targeting by autophagy^35^, and it has been found that the autophagy machinery targets broken BCV membranes^23^. Therefore, it was interesting to observe a significant increase in the intracellular bacteria that could not unwrap from the BCV remnants being associating with canonical autophagic markers including p62 (Supplementary Fig. 2C), NDP52 (Supplementary Fig. 2D) and LC3 (Fig. 2E) in p150^Glued^-depleted condition. Taken together, these observations implicated that dynein plays a role in the disintegration of *Shigella* BCV upon BCV damage, thereby encouraging the intracellular motility of the bacteria and avoiding canonical LC3-dependent autophagic signaling.

### Activating adaptors Ninein-like (NINL) and BICD family-like cargo adapter 2 (BICDL2) regulate dynein-mediated BCV disintegration

The dynein/dynactin complex requires activating adaptors to move processively on microtubules. These adaptors have been proposed to play a role in dictating the cargoes to be carried by dynein^11,36^. We thus depleted individual activating adaptors in HeLa cells by RNA interference and employed our time-lapse quantitative microscopic assays to evaluate: (i) how quickly the BCV membranes moved away from *Shigella*, and (ii) the time taken for the bacteria to acquire intracellular motility. With these experiments, we aimed at elucidating the molecular mechanism of dynein-mediated BCV disintegration during *Shigella* infection.

**Fig. 3:**
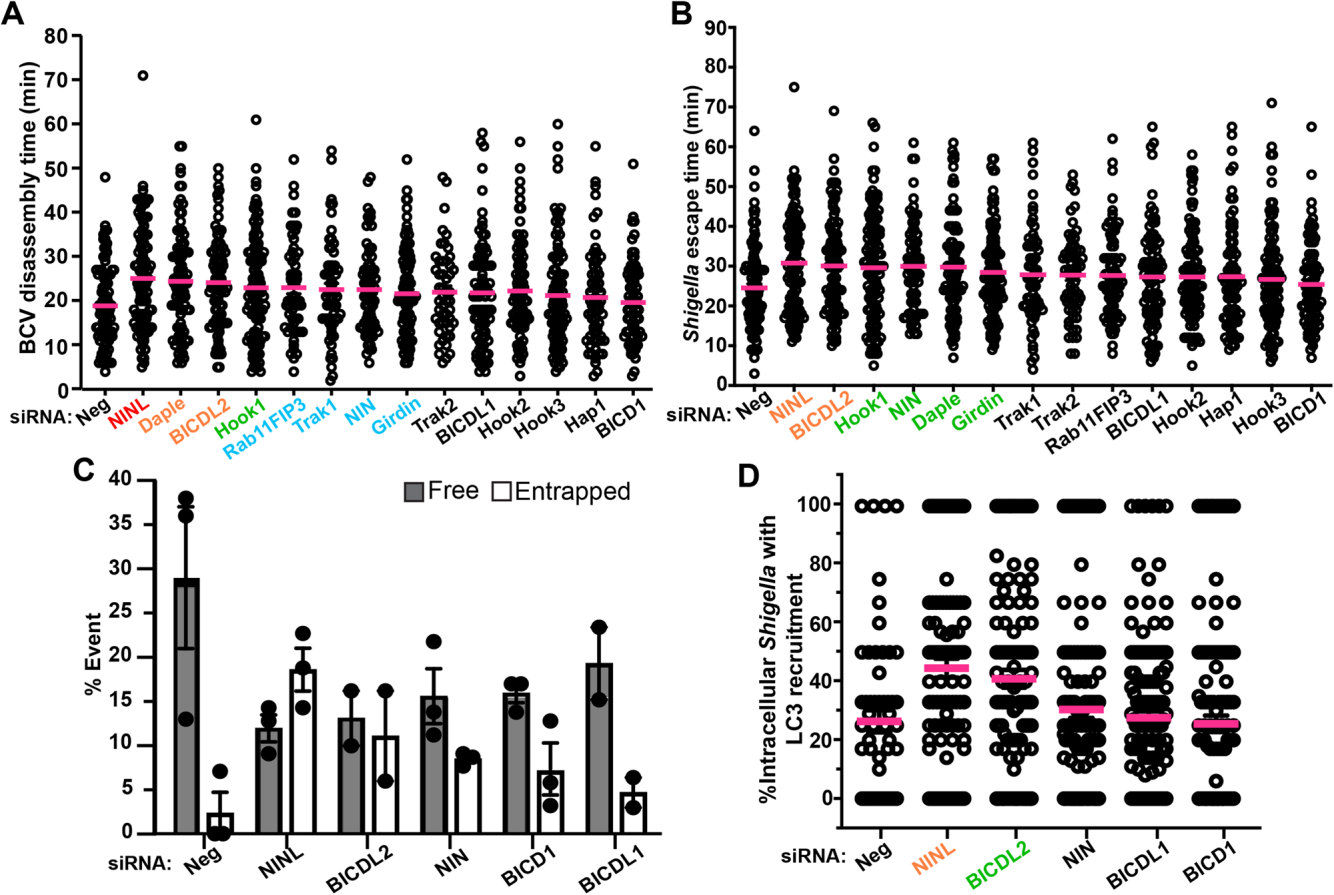
Activating adaptors NINL and BICDL2 regulate dynein-mediated BCV disintegration. Time-lapse microscopic analyses of *Shigella* infection of cells subjected to RNA interference of non-targeting control (siRNA Neg) versus depletion of dynein activating adaptors were performed to examine the **A** time of BCV disassembly and **B** *Shigella* escape time. *n* > 50 infected cells in three independent replicates in each condition. The data were presented in the descending order of the average times determined in the respective assays (left to right). The bars (magenta) represent the mean and two-tailed Welch’s t-tests were performed. (The colors of the x-axis labels indicate the statistical significance - red: ****p < 0.0001; orange: ***p < 0.001; green: **p < 0.01; blue: *p < 0.05; black: non-significant). **C** Analysis of the fates of individual *Shigella* BCVs in control HeLa cells and cells subjected to depletion of the subset of dynein activating adaptors that showed the strongest effects determined by the analyses in **A**, **B**. The bars represent mean ± SEM of at least two independent replicates in each condition. **D** Analysis of the recruitment of LC3 to intracellular *Shigella* in infected cells at 45 min-post infection in cells subjected to RNA interference of non-targeting control (siRNA Neg) versus depletion of the subset of dynein activating adaptors. *n* > 60 infected cells in three independent replicates in each condition. The bars (magenta) represent the mean and two-tailed Welch’s t-tests were performed. (The colors of the x-axis labels indicate the statistical significance – orange: ***p < 0.001; green: **p < 0.01). Source data are provided as a Source Data file.

Among the 14 activating adaptors tested, we found that depletion of ninein-like protein (NINL), Daple (also called CCDC88C) and BICD family-like cargo adapter 2 (BICDL2) showed the strongest effects on interrupting the time of BCV disassembly (Fig. 3A). Interestingly, the strongest delay in *Shigella* actin-based motility was also observed in NINL and BICDL2 depleted cells (Fig. 3B). After this preliminary screen, we further analyzed the role of dynein activating adaptors on individual *Shigella* and their BCVs upon BCV perforation. We observed more than a 2-fold decrease in *Shigella* moving free of BCV membranes in NINL- or BICDL2-depleted cells ( ~ 12% in NINL- or BICDL2-depleted conditions while ~30% in control) (Fig. 3c). There was a more than 4-fold increase in the number of *Shigella* being entrapped in the damaged BCVs in NINL-depleted ( ~ 19%) or BICDL2-depleted ( ~ 11%) condition when compared with the control (2%) (Fig. 3C). We further demonstrated a significant increase in the proportion of LC3-associated intracellular *Shigella* when NINL or BICDL2 was depleted ( > 40% intracellular *Shigella* associated with LC3 in NINL- and BICDL2-depleted condition compared to only ~25% in control) (Fig. 3D). These data are consistent with our observations in p150^Glued^-depleted cells. Of note, the interference of ninein (NIN), the close family member of NINL, or other members of the BICD family (namely BICD1 and BICDL1) displayed negligible effects in all the assays tested, similar to cells treated with scrambled siRNA (compare with negative control). Using immunofluorescence staining, we confirmed the recruitment of NINL and BICDL2 to *Shigella* infection foci (possibly on the IAMs) at 30 min post-infection (Supplementary Fig. 3A, B, respectively). These results implicated that dynein-mediated *Shigella* BCV unwrapping is possibly regulated via specific interaction with the activating adaptor NINL and BICDL2.

### Rab13 is recruited to the *Shigella* BCV via dynein activating adaptor BICDL2

We wanted to understand the molecular pathways subverted by *Shigella* for the unwrapping of BCV remnants through the dynein complex. In this context, we took note that NINL is linked to Rab8-mediated vesicular trafficking^37,38^, whereas BICDL2 is reported to interact strongly with Rab13 and some other Rab GTPases in weaker affinities^39^. Furthermore, coordinated actions of Rab8 and Rab13 have been reported to regulate the shuttling of cargoes that are critical to the establishment of adherent junctions and tight junctions^40^, and Rab13 has also been implicated in membrane ruffling and macropinosome formation in macrophages^41^. On the other hand, during *Shigella* invasion we have identified Rab8 being significantly enriched on *Shigella* IAMs (Supplementary Table 1)^24^. We observed the localization of NINL and BICDL2 on some membrane compartments that were decorated with their interacting Rab GTPases during *Shigella* infection (Supplementary Fig. 3C, D, respectively). Interestingly, Rab13 and BICDL2 were also found to be localized in close proximity to the bacteria (Supplementary Fig. 3D), indicating that Rab13 and BICDL2 might be present in the *Shigella* BCV.

**Fig. 4:**
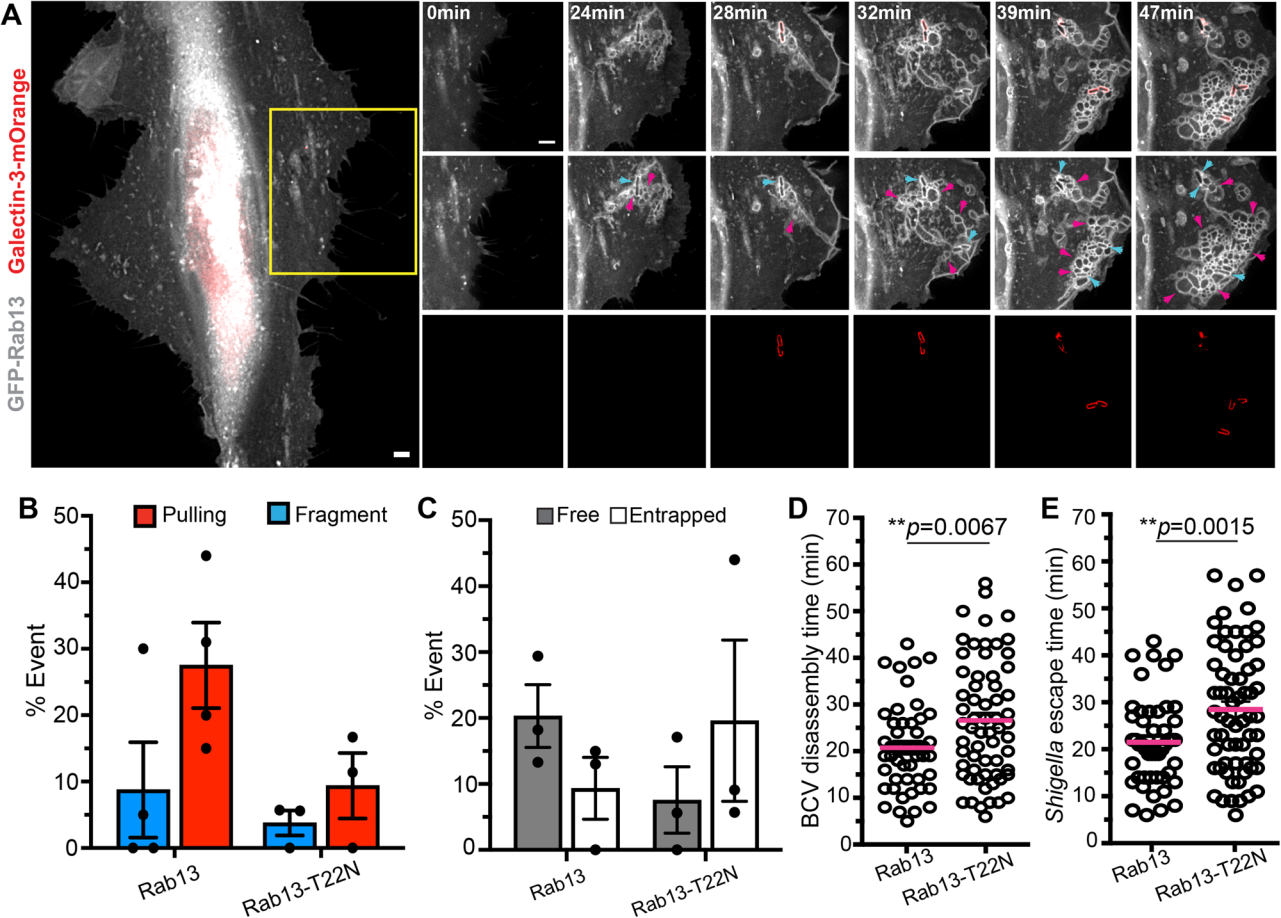
Rab13 is recruited to *Shigella* BCV for membrane translocation. **A** Time-lapse microscopic images of the recruitment of Rab13 (gray) in transfected HeLa cells. Galectin-3-mOrange (red) was used as a marker of vacuolar rupture. Images were captured every minute and maximum z-projection of the representative infection focus is shown. Blue arrowheads indicate the localization of Rab13-positive BCV while the magenta arrowheads indicate some Rab13-positive infection-associated macropinosome. Scale bars are 5 μm. **B** Analysis of BCV membrane pulling or fragmentation as illustrated in Fig. 1B, C in cells expressing Rab13 and the GDP-mimicking mutant Rab13-T22N. The bars represent mean ± SEM of at least three independent replicates. **C** Analysis of the fates of individual *Shigella* BCVs in cells expressing Rab13 or Rab13-T22N. The bars represent mean ± SEM of three independent replicates. Analysis of the **D** time of *Shigella* BCV disassembly and **E** *Shigella* escape time in cells expressing Rab13 or Rab13-T22N. n > 50 infected cells in three independent replicates. The data are presented as mean(magenta bars) ± SEM and two-tailed Welch’s t-tests were performed (**p < 0.01). Source data are provided as a Source Data file.

The involvement of Rab13 in *Shigella* invasion has never been studied, despite ample support for Rab5, Rab8, and Rab11 recruitment to *Shigella* IAMs^40^. To our surprise, we discovered that in addition to the other Rabs, we found Rab13 on the ruffles induced upon *Shigella* invasion by time-lapse microscopy, and Rab13 was enriched on *Shigella* IAMs and the BCV before BCV perforation. Particularly pertinent was the localization of Rab13 on the membrane remnants that moved away from the bacteria (Fig. 4A and Supplementary Movie 6). Furthermore, the constitutively inactive mutant of Rab13, Rab13-T22N, no longer localized on *Shigella* IAMs nor BCV, indicating that Rab13 recruitment to BCV depends on its nucleotide status (Supplementary Fig. 4). This observation prompted us to investigate possible connections with Rab13 and our data that implicated the dynein motor in membrane disassembly.

To decipher the role of Rab13 in *Shigella* invasion, we focused on the conspicuous “pulling” or “fragment” phenotypes of BCV remnants as illustrated in Fig. 1B, C which we identified to be associated with the dynein motor complex. We first infected wild-type Rab13- or Rab13-T22N-expressing cells (which does not associate with the BCV), and we traced the dynamics of BCV membrane disassembly in these cells. We observed about a 3-fold higher occurrence of “pulling” and “fragment” phenotypes in Rab13-expressing cells compared to Rab13-T22N (Fig. 4B), indicating that the BCV membrane remnants are more actively carried away when Rab13 is functional. We further found that about 10% of *Shigella* moved within the damaged BCV in the acquired movies (Fig. 4C, “Entrapped”), whereas about 20% of the bacteria started intracellular motility without any BCV membrane remnants (Fig. 4c, “Free”) in Rab13-expressing cells. In contrast, a 2-fold increase ( ~ 20%) in damaged BCV-entrapped *Shigella* (Fig. 4C, “Entrapped”) and a 2.5-fold decrease in *Shigella* moving free ( ~ 7.5%) (Fig. 4C, “Free”) were observed in Rab13-T22N-expressing cells. These observations substantiate the reduced membrane dynamics and a higher probability of *Shigella* being confined inside a vacuole in the presence of the functionally-dead Rab13 mutant (Rab13-T22N). We further verified a significant delay in BCV disintegration (26.6 ± 2.1 min) (Fig. 4D), in conjunction of the prolonged time for *Shigella* to completely escape from its vacuole (28.5 ± 2.1 min) (Fig. 4E) in Rab13-T22N-expressing cells, when compared with Rab13-expressing cells (an average of 20 min for both BCV disassembly and *Shigella* escape). These analyses clearly corroborate the notion that Rab13 is involved in the efficient membrane movements of *Shigella* BCV remnants, similar to our data on dynein and its activating adaptors BICDL2 and NINL, thereby promoting the intracellular motility of the bacteria.

### *Shigella* T3SS effector IpaH7.8 mediates Rab13 recruitment and retention on *Shigella* BCV remnants

**Fig. 5:**
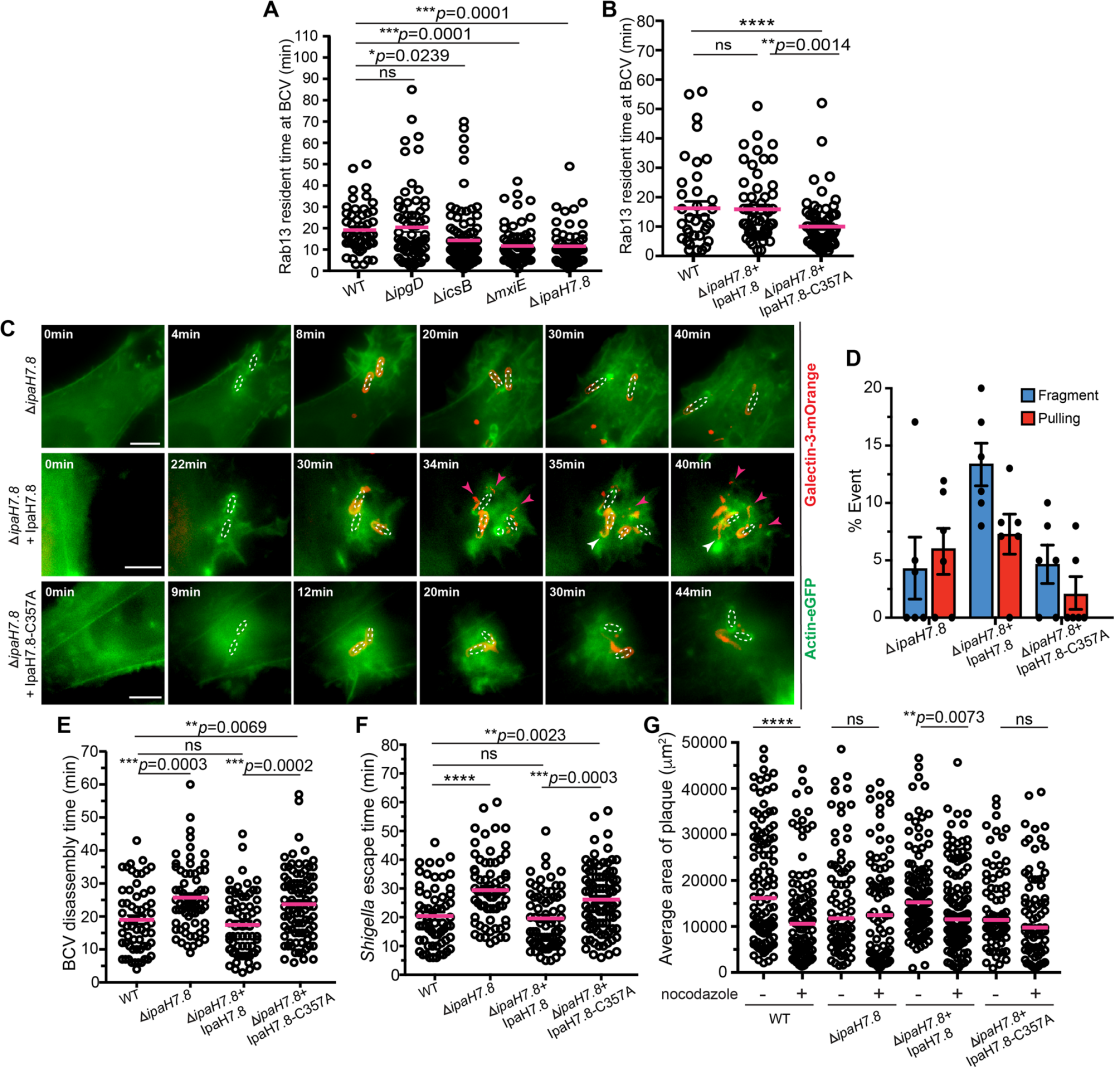
*Shigella* T3SS effector IpaH7.8 is involved in recruiting Rab13 to *Shigella* BCV via its ubiquitin E3 ligase activity. **A** Time of Rab13 residence at *Shigella* BCV in the presence of wild-type and different *Shigella* mutants. *n* > 50 infected cells in three independent replicates. The bars (magenta) represent the mean. **B** Time of Rab13 residence at *Shigella* BCV in the presence of *Shigella* wild-type, *ipaH7.8* mutant complemented with wild-type IpaH7.8 (Δ*ipaH7.8*+IpaH7.8) or *ipaH7.8* mutant complemented with IpaH7.8-C357A mutant (Δ*ipaH7.8*+IpaH7.8-C357A). n > 35 infected cells in three independent replicates. The data are presented as mean(magenta bars)±SEM. **C** Microscope analysis of the BCV disassembly status in cells infected with Δ*ipaH7.8* (top panel), Δ*ipaH7.8*+IpaH7.8 (middle panel), or Δ*ipaH7.8*+IpaH7.8-C357A (bottom panel). Images were captured every minute. Maximum z-projections of the representative infection foci are shown. Scale bars are 5 μm. Pink arrowheads indicate the moving pieces of the BCV membrane. White arrowheads indicate the sites of membrane pulling. **D** Analysis of BCV membrane pulling or fragmentation (as illustrated in Fig. 1B, C) in cells infected with different *Shigella* mutants. The bars represent mean ± SEM of six independent replicates. Analysis of the **E** time of *Shigella* BCV disassembly and **F** *Shigella* escape time in cells infected with wild-type and different *Shigella* mutants. *n* > 60 infected cells in three independent replicates in each condition. The bars (magenta) represent the mean. **G** Analysis of sizes of infection plaques in Caco-2/TC7 cells infected with wild-type and different *Shigella* mutants in the absence or presence of nocodazole at 18 hrs post-infection. *n* > 70 plaques in three independent replicates in each condition. The bars (magenta) represent the mean. Statistical analysis in Fig. 5 used two-tailed Welch’s t-test, with reported *p*-values for significance comparison (ns: non-significant; *p < 0.05; **p < 0.01; ***p < 0.001; ****p < 0.0001). Source data are provided as a Source Data file.

By quantitative analysis of our time-lapse data, we investigated for how long Rab13 remained recruited and localized on the BCV enclosing the intracellular *Shigella*. We determined that Rab13 resided on *Shigella* BCV membranes for an average of 20 min (Fig. 5A). We next analyzed by screening a collection of *Shigella* T3SS effector mutants whether bacterial factors are involved in retaining Rab13 at the BCV, and whether this had an impact on the BCV unpeeling process. To study this, we first measured the residence time of Rab13 on BCV membranes depending on the specific *Shigella* T3SS effectors. So far, the *Shigella* T3SS effector IpgD had already been demonstrated to mediate the recruitment of Rab8 and Rab11 to *Shigella* IAMs^20,24,42^. However, the *Shigella ipgD* mutant showed no effect on Rab13 residence on the BCV (20.4 ± 2.6 min) despite its interaction with Rab8, a Rab GTPase that is recognized as the same family as Rab13 (Fig. 5A). Another report on the *Shigella* effector IcsB had suggested that it modifies a cohort of host proteins, including Rab13, by its acyltransferase activity to possibly enhance membrane anchor of the modified proteins^43^. We thus also tested the Rab13 recruitment in the presence of the *icsB* mutant and found that Rab13 stayed for a shorter time at the BCV compared to the infection of the WT strain (14.3 ± 2.2 min) (Fig. 5A).

Intriguingly, we found that Rab13 resided for the shortest time when infected with *Shigella mxiE* mutant (11.7 ± 1.9 min) (Fig. 5A). MxiE is an AraC-like family transcriptional regulator that promotes the expression of a subset of *Shigella* effectors that include the IpaHs, a family of related proteins that are E3 ubiquitin ligases^44^. Among the IpaHs, IpaH7.8 has been shown to promote *Shigella* vacuolar escape in macrophages^45^. We tested if members of IpaH family plays a role in governing the recruitment of Rab13 during *Shigella* infection. IpaH1.4, IpaH4.5 and IpaH9.8 showed no observable effects on the residence time of Rab13 on *Shigella* BCV membranes when infecting HeLa cells with those bacterial mutants (Supplementary Fig. 5A). In contrast, the *ipaH7.8* mutant exhibited a significant decrease in the Rab13 residence on BCV, similar to that observed for the *mxiE* mutant (11.6 ± 2.0 min) and stronger than the effect measured for IcsB (Fig. 5A). Complementing the wild-type allele of IpaH7.8 to *ipaH7.8* mutant bacteria restored Rab13 residence time to the wild-type *Shigella* condition (15.9 ± 3.0 min), whereas complementation of the catalytically inactive IpaH7.8-C357A not exhibiting any E3 ligase activity^46,47^ to our *ipaH7.8* mutant bacteria failed to impose such a recovery (10.0 ± 2.8 min) (Fig. 5B). Membrane fragmentation and pulling were also more apparent in the *ipaH7.8* mutant complemented with the wild-type IpaH7.8 (Fig. 5C, middle panel) than in the *ipaH7.8* mutant (Fig. 5C, top panel) or in *ipaH7.8* mutant complementing IpaH7.8-C357A (Fig. 5C, bottom panel). We analyzed the BCV disassembly status in the same way as in Fig. 1B, and we found that membrane fragmentation and pulling events were more frequently observed in *ipaH7.8* mutant complemented with the wild-type IpaH7.8 (Fig. 5D). The reduced events of the BCV disassembly in *ipaH7.8* mutant or in *ipaH7.8* mutant complementing IpaH7.8-C357A were highly reminiscent of the conditions when Rab13 was functionally dead (Fig. 4B). We further demonstrated that only complementing the wild-type IpaH7.8 to a *mxiE* mutant was able to fully rescue the phenotype, but not the catalytically inactive IpaH7.8-C357A (Supplementary Fig. 5A). By immunofluorescence, both IpaH7.8 and IpaH7.8-C357A were enriched at *Shigella* infection foci and were localized very close proximity to the bacteria (Supplementary Fig. 5C, D) (as shown by quantification in Supplementary Fig. 5E). Taken together, these data show that the E3 ubiquitin ligase activity of IpaH7.8 plays a role in recruiting or retaining Rab13 to *Shigella* BCV for efficient membrane unpeeling.

We next quantified the effects of the *ipaH7.8* mutant during vacuolar escape showing a significant delay in full BCV disassembly (25.7 ± 1.3 min) (Fig. 5E), which correlated with the delay in *Shigella* vacuolar escape via an actin tail (29.4 ± 2.0 min) (Fig. 5F). These findings were in agreement with a previous report that the phagosomal escape of the bacteria was hindered in the absence of IpaH7.8 while invasion was not affected^48^. Complementing the wild-type IpaH7.8 to the *ipaH7.8* mutant completely rescued both phenotypes (17.4 ± 1.2 min for BCV disassembly and 19.6 ± 1.2 min for the initiation of actin-based motility) (Fig. 5E, F). In contrast, complementing the catalytically inactive IpaH7.8-C357A to the *ipaH7.8* mutant bacteria showed apparent obstruction in the BCV disassembly (23.7 ± 1.1 min) (Fig. 5E) and infection progression via an actin tail (26.1 ± 1.2 min) (Fig. 5F) as in the *ipaH7.8* mutant, suggesting that the ubiquitin ligase activity of IpaH7.8 correlated strongly with the phagosomal escape of *Shigella*. Intriguingly, infecting HeLa cells with the *mxiE* mutant or the *ipaH7.8* mutant in the presence of the proteasomal inhibitor MG132 could not revert the reduced Rab13 residence time on *Shigella* BCV (Supplementary Fig. 5B).

The ability of *Shigella* to disseminate intercellularly is essential for its virulence. We thus further investigated the effect of impaired BCV uncoating on cell-to-cell spread of *Shigella* by measuring the sizes of plaques formed on the cell monolayer after 18 h. We found that the sizes of plaques formed by wild-type *Shigella* were significantly reduced in the presence of the microtubule-depolymerization drug nocodazole (Fig. 5G). This phenotype was also observed in the *ipaH7.8* mutant complemented with the wild-type IpaH7.8 (Fig. 5G). On the contrary, the plaques formed by bacterial strains that exhibited inhibited BCV uncoating (i.e. *ipaH7.8* mutant or *ipaH7.8* mutant complementing with the catalytically inactive IpaH7.8-C357A) were apparently smaller than that of the wild-type strain, whereas these strains were also insensitive to the drug treatment (Fig. 5G). Collectively, our findings suggest a proteasome-independent role for the catalytic activity of IpaH7.8 in promoting Rab13 recruitment to the BCV and in the manipulation of the host microtubule-based transport for efficient vacuolar escape by *Shigella*.

## Discussion

Microbial pathogens subvert the cytoskeletal transport tracks of the host during different stages of microbial pathogenesis. We deciphered the clear preference for microtubule-based transport routes by the bacterial pathogen *Shigella* for efficient membrane uncoating, cytosolic colonization, and intercellular spread. Specifically, we uncovered the hijacking of the microtubule-based dynein motor and its regulatory Rab GTPases, Rab8 and Rab13, in NINL- and BICDL2-dependent manner in order to facilitate the displacement of membrane remnants from the intracellular bacteria. We further elucidated that the *Shigella* bacterial effector IpaH7.8 plays an important role in this cascade regulating Rab13 recruitment and retention on the membrane of *Shigella* containing vacuoles and damaged BCV membranes.

Viruses have been shown in previous reports to manipulate dynein during different stages of their infection cycle^11,49,50^. On the other hand, little has been documented on whether bacterial microbes also employ dynein for host-pathogen interactions^51^. The unique functions of dynein are tightly regulated by the activating adaptors to differentiate the types of cargoes to be carried and to initiate the motion of dynein. In this work, we provided strong evidence that dynein plays a significant role in displacing *Shigella* BCV membrane remnants (Fig. 2). Membrane uncoating is important for the pathogen to move efficiently via an actin tail as well as avoiding the targeting by the host autophagy machinery to the damaged BCV membranes. Our data further suggested that dynein activating adaptors NINL and BICDL2 were specifically involved, but not the other activating adaptors in our tested conditions (Fig. 3A–D). Although Rab11 has been reported to be hijacked by *Shigella* for BCV rupture^20,42^, Rab11FIP3, a Rab11-related activating adaptor, showed only trivial effects on *Shigella* BCV rupture and fragmentation (Fig. 3A). One possible implication is that Rab11 possesses other dominating functional roles (e.g. recruiting the exocyst to cluster IAMs for BCV-IAM interaction as reported recently, etc.)^24^ during *Shigella* invasion rather than dynein regulation. Another possibility is that dynein involvement was completely independent of Rab11. This suggests a multi-step process of vacuolar escape through a first step of BCV damage (involving Rab11) followed by BCV membrane unpeeling (through the Rab13-dynein-microtubule cascade).

The identification of the precise involvement of NINL and BICDL2 in our unbiased screen provides an important clue for revealing the regulatory mechanism of dynein by Rab8 and Rab13. We also elucidated the IpaH7.8-regulated Rab13 trafficking facilitating BCV disintegration. Previously, Rab8 and also Rab11 are targeted through the effector IpgD^20,24^. Together, this previous data and our new results expand the repository of host Rab GTPases that are subverted to promote infection progression and intracellular survival of bacterial pathogens. It remains to be investigated in the future whether Rab8 and Rab13 are involved in a coordinated cascade or whether they represent two independent or successive trafficking pathways to ensure maximal efficiency in membrane damage followed by uncoating for cytosolic access of the pathogen.

Many bacterial pathogens use the host ubiquitin system to remodel phagosomes and modulate the host pathways^52–55^. Our work disclosed the intimate role of the E3 ubiquitin ligase activity of IpaH7.8 on Rab13 residence time on *Shigella* BCVs (Fig. 5A, B). Of note, the IpaH7.8-Rab13 interaction appeared to be unique among other IpaH family members with no functional redundancy by other bacterial effectors (Supplementary Fig. 5A). Hence, our data not only confirm the previously proposed role of IpaH7.8 in phagosomal escape of *Shigella*^48^, but also provide strong evidence in uncovering the functional implications of IpaH7.8 for such a process. The precise mechanism by which Rab13 is being retained by IpaH7.8 remains to be elucidated. One hypothesis is that IpaH7.8 induces ubiquitination of Rab13 to modulate its localization on membranous compartments. One example is the *Salmonella* effector protein SopB that shuttles between the plasma membrane and the *Salmonella* vacuole depending on its ubiquitination status, implying that ubiquitination of a protein might differentiate its membrane localization^56^. Interestingly, SopB is also involved in controlling *Salmonella* vacuolar integrity in epithelial cells, however this takes place through a mechanism of vacuolar size control^34^. During *Legionella pneumophila* infection, a scenario has been proposed where several Rab GTPases localized on the *Legionella*-containing vacuole are ubiquitinated possibly through the action of multiple *Legionella* effector ubiquitin ligases with an unclear mechanism^57^. An alternative hypothesis is that IpaH7.8 ubiquitinates yet-to-be-determined factor(s) that indirectly drive(s) the recruitment and retention of Rab13 on *Shigella* vacuole. This latter hypothesis is attractive, given that the membrane anchor of Rab13 has been proposed to be directed by protein-protein interactions but not prenylation at its C-terminus^58^. IpaH7.8 has been reported to target gasdermins during *Shigella* invasion, however, it appears unlikely that this plays a role in the cascade involving Rab13^59,60^. Validations of additional interacting partners and the ubiquitination candidates of IpaH7.8 are currently under investigation by our team to clarify these hypotheses.

We validated the substantial role of dynein in *Shigella* pathogenesis in the present work, whereas previously we discovered a considerable enrichment of many kinesin motors on *Shigella* IAMs (Supplementary Table 1)^24^. A growing body of research suggests a functional consequence for the colocalization of both dynein and kinesins on the same membranous cargo^8,10^. Our findings are congruent with previous studies in adenovirus, where the dynein motor acts to deliver the newly entered virus to the nuclear pore. Subsequent recruitment of kinesin motors to the virus supplies the opposing force to the docked virus. These forces act to disassemble the viral capsid in a manner strikingly similar to that observed by microtubule motors acting on the *Shigella* BCV^18^. Although the precise roles and dynamics of these microtubule-based molecular motors in microbial pathogenesis of *Shigella* need further study, it is tempting to speculate that the disproportionate mechanical forces exerted by different molecular motors may promote the fragmentation of the perforated BCV and hence the efficient unwrapping of the BCV membrane. In this context, the subversion of the microtubule network by *Shigella* may be conducive to establish a favorable microenvironment for BCV-IAMs clustering and interaction to facilitate such an unwrapping mechanism for vacuolar escape. Our work thus highlights the new paradigm of the subversion of host cytoskeletal transport by microbial pathogens for establishing its intracellular replicative niche.

## Methods

Reagents and resources used in this study are listed in Supplementary Data 1.

### Cell culture and transfection

HeLa cells were cultured in DMEM supplemented with 10% fetal bovine serum (FBS) at 37 °C in the presence of 5% CO_2_. Transfection of plasmids was performed using FuGENE Transfection Reagent (Promega) according to the manufacturers’ protocol. The sequences of siRNA for p150Glued are obtained from Eurogentec: GGUAUCUGACACGCUCCU and UAGGAGCGUGUCAGAUAC. Specific ON-TARGETplus Smartpool siRNAs against dynein activating adaptors were obtained from Dharmacon, GE Healthcare: NIN (L-019133-0-0005), NINL (L-018162-01-0005), BICD1 (L-019496-00-0005), BICDL1 (L-027129-02-0005), BICDL2 (L-022613-02-0005), Hook1 (L-016845-01-0005), Hook2 (L-020408-02-0005), Hook3 (L-013558-01-0005), Daple (L-033364-01-0005), Girdin (L-032517-02-0005), Rab11FIP3 (L-021079-02-0005), Trak1 (L-020331-01-0005), Trak2 (L-014141-00-0005), Hap1 (L-011560-00-0005). Non-targeting siRNA ON-TARGETplus (D-001810-10-05) (Dharmacon, GE Healthcare) served as the siRNA control. siRNA transfection was performed using Lipofectamine RNAiMAX transfection reagent (Thermo Fisher) at a final concentration of 40 nM for 72 h before infection. Protein knock-down efficiency was confirmed by real-time quantitative PCR.

### Bacterial strains and infection

*Shigella* strains used in this study, including the wild-type *S. flexneri* serotype M90T strain (M90T-AfaI), blue fluorescent protein-expressing M90T, Δ*ipgD* mutant (Δ*ipgD*), Δ*ipaH7.8* mutant (Δ*ipaH7.8*), Δ*icsB* mutant (Δ*icsB*), and *mxiE* mutant (*mxiE::kan*) express the adhesion *afaI*. Δ*ipaH1.4* mutant (Δ*ipaH1.4*), Δ*ipaH4.5* mutant (Δ*ipaH4.5*), Δ*ipaH9.8* mutant (Δ*ipaH9.8*), Δ*ipaH7.8* mutant complementing wild-type IpaH7.8 (Δ*ipaH7.8* + IpaH7.8) and Δ*ipaH7.8* mutant complementing enzymatic dead IpaH7.8-C357A mutant (Δ*ipaH7.8* + IpaH7.8-C357A) required poly-L-lysine treatment at room temperature for 15 min prior to the infection experiment as described before^42^. *Salmonella enterica* serovar Typhimurium SL1344 wild-type strains were used in this study. For *Shigella* culture, all bacterial strains were grown in TCS medium supplemented with 100 μg/mL ampicillin at 37 °C. For *Salmonella* culture, bacteria were grown in LB medium supplemented with 100 μg/mL ampicillin at 37 °C. On the day of infection, *Shigella* strains were subcultured in 1:100 dilution in TCS medium supplemented with 100 μg/mL ampicillin at 37 °C until an OD_600_ of ~0.5. *Salmonella* strain was subculture in 1:50 dilution in LB medium supplemented with 100 μg/mL ampicillin and 0.3 M NaCl at 37 °C until an OD_600_ of ~2.0. All bacterial strains were harvested using centrifugation at 8000 *g* for 1 min and then washed once in EM buffer (25 mM HEPES, pH 7.3, 120 mM NaCl, 7 mM KCl, 1.8 mM CaCl_2_, 0.8 mM MgCl_2_, 5 mM glucose). Bacteria were resuspended in EM buffer and diluted to MOI 20 for fixed experiment or MOI 50 for live experiment. Unless specified, for the treatment with microtubule-depolymerizationdrugnocodazole or taxol, HeLa cells were treated with nocodazole (16 μM) or taxol (10 μM) for 2 hr. For the treatment with proteasome inhibitor MG-132 (50 μM), the inhibitor was complemented to the medium during bacterial infection. HeLa cells were washed with warm EM buffer 3 times prior to bacterial infection. Bacterial infection was performed in the presence of the chemical inhibitors. Cells treated with 0.01% DMSO for the same incubation period served as control.

### Time-lapse microscopy

6000 cells per well were seeded in 4-well inserts (Ibidi-#80649) in glass-bottom dishes (Ibidi-#81158) three days prior to infection experiment. Plasmid transfection was performed the following day using FuGENE HD transfection reagent (Promega) for 48 h. On the day of infection, cells were washed with warm EM buffer for three times. Then, cells were challenged with the bacteria at a MOI of 50. Live imaging was performed on DeltaVision Elite (GE Healthcare) using Olympus 60×/1.42 NA oil objective with refractive index oil 1.520 (GE Healthcare). Images were recorded every 30 s for imaging microtubule dynamics for 90 min or every minute for other experiment for 120 min at a step-size of 0.3 μm in z-plane at 37 °C. Images were processed using the built-in deconvolution analysis module.

### Recruitment of autophagic markers to intracellular bacteria

HeLa cells expressing galectin-3-mOrange (transfected with siRNA against p150^Glued^ or scramble control) were infected with *Shigella* at an MOI of 20 at 37 °C. Bacterial infection was performed as described above. After 45-min infection, samples were washed three times with ice-cold PBS and were fixed using 4% paraformaldehyde at room temperature for 15 min. Samples were permeabilized with 0.05% saponin in blocking buffer (20% fetal bovine serum in PBS) at room temperature for an hour. For immunofluorescence staining, samples were incubated with the anti-rabbit anti-LC3 antibody (Abcam, #ab48394) or rabbit anti-p62 (MBLbio, #PM045), or rabbit anti-NDP52 (Abcam, #ab68588) in 1:200 dilution in the presence of dilution buffer (0.05% saponin, 2% bovine serum albumin in PBS) at room temperature for an hour. Samples were washed three times with PBS prior to secondary anti-rabbit conjugated to Alexa Fluor 488 dye (Invitrogen, #A11034) in 1:500 dilution at room temperature for an hour. Cell nuclei and bacteria were stained with DAPI (1 ng/mL) (Thermo Fisher) at room temperature for 20 min. 10 images were acquired under a Perkin Elmer Ultraview spinning disk confocal microscope using a 60×/1.2 NA water objective at a step-size of 0.3 μm in z-plane.

### Infection plaque assay

Cell monolayers were prepared by seeding 25000 Caco-2/TC7 cells in 96-well plates 2 days prior to the experiment. On the day of infection, cells were washed with warm EM buffer three times. Then, cells were infected with the wild-type or mutant strains at a MOI of 0.5 in the absence or presence of 1 mM nocodazole. After 1-h infection, cells were washed 3 times with warm EM buffer. Cells were then incubated with EM buffer supplemented with 100 μg/mL gentamicin in the absence or presence of 1 mM nocodazole for an hour. After that, cells were incubated with DMEM buffer supplemented with 10% FBS and 50 μg/mL gentamicin in the absence or presence of 1 mM nocodazole overnight. At 18 h post-infection, cells were washed three times with ice-cold PBS and were fixed with 4% paraformaldehyde at room temperature for 15 min. Samples were permeabilized with 0.05% saponin in blocking buffer (1% bovine serum albumin, 10% fetal bovine serum in PBS) at room temperature for an hour. For immunofluorescence of intracellular *Shigella*, samples were incubated with anti-rabbit anti-*Shigella* antibody (Abcam, #ab65282) in 1:200 dilution. Samples were washed three times with PBS and then incubated with the secondary anti-rabbit conjugated to Alexa Fluor 488 dye (Thermo Fisher, #A11034) in 1:500 dilution. Phalloidin conjugated to Alexa Fluor 647 (Thermo Fisher, #A22287) was used to label actin, and DAPI (Thermo Fisher, #62248) to label cell nuclei at room temperature for 20 min. Images were acquired under a LSM800 confocal microscope using a 20×/0.8 NA air objective or under a Perkin Elmer Ultraview spinning disk confocal microscope using a 20x/0.75 NA air objective at a step-size of 0.9 μm in z-plane. Images were analyzed in Fiji with a maximum Z-projections of the images. Discrete plaques formed on the cell monolayer were outlined with reference to the cell boundaries marked by actin using the freehand selection in Fiji and the areas of the plaques were measured.

### Immunofluorescence

Infection was performed as described above. Samples were fixed using 4% paraformaldehyde at room temperature for 15 min. For immunofluorescence staining, the cells were permeabilized using 0.25% saponin in blocking buffer (1% bovine serum albumin, 10% fetal bovine serum in PBS) at room temperature for an hour prior to primary antibody staining. Primary and secondary antibodies staining were performed in the presence of 0.05% saponin in blocking buffer (1% bovine serum albumin, 10% fetal bovine serum in PBS) at room temperature for an hour. For immunofluorescence of endogenous dynein, samples were incubated with the primary antibody using anti-mouse anti-DYNC1I1 antibody (Sigma-Aldrich, #MAB1618) in 1:200 dilution. For immunofluorescence of FLAG-tagged proteins, samples were incubated with primary antibody using anti-mouse FLAG-M2 antibody (Sigma-Aldrich, #F3165) in 1:2000 dilution. For immunofluorescence of endogenous BICDL2, samples were incubated with the primary antibody using anti-rabbit anti-BICDL2 antibody (Thermo Fisher, #PA5-60293) in 1:200 dilution. Samples were washed three times with PBS prior to secondary anti-mouse conjugated to FITC (Thermo Fisher, #F-2761) or Cyanine3 (Thermo Fisher, #A10521) or anti-rabbit conjugated to Alexa Fluoro 488 (Thermo Fisher, #A11034) or Cyanine3 (Thermo Fisher, #A10520) in 1:500 dilution. Cell nuclei and bacteria were stained with DAPI (1 ng/mL) (Thermo Fisher) and actin foci were labeled by rhodamine-phalloidin (Thermo Fisher) at room condition for 20 min. Images were acquired under a Perkin Elmer Ultraview spinning disk confocal microscope using a 60×/1.2 NA water objective at a step-size of 0.3 μm in z-plane.

### Statistical analysis

Statistical analyses were performed using the software GraphPad Prism v8. Two-tailed Welch’s t-test was performed, where *p* < 0.05 was considered as statistically significant: *p < 0.05, **p < 0.01, ***p < 0.001, ****p < 0.0001.

### Reporting summary

Further information on research design is available in the Nature Portfolio Reporting Summary linked to this article.

### Supplementary information

Supplementary Information

Peer Review File

Supplementary Movie 1

Supplementary Movie 2

Supplementary Movie 3

Supplementary Movie 4

Supplementary Movie 5

Supplementary Movie 6

Supplementary Data 1

Reporting Summary

### Source data

Source Data

## Data Availability

The data that support the findings of this study are available within the article and supplementary information. Source data are provided in this paper.
