# Supplementary material for: Microtubules provide force to promote membrane uncoating in vacuolar escape for a cyto-invasive bacterial pathogen
Source: Nat Commun. 2024 Feb 5;15:1065. doi: 10.1038/s41467-024-45182-6 (PMC10844605; doi:10.1038/s41467-024-45182-6)
Supplement: Supplementary file 1 — Supplementary Information [file 41467_2024_45182_MOESM1_ESM.pdf]

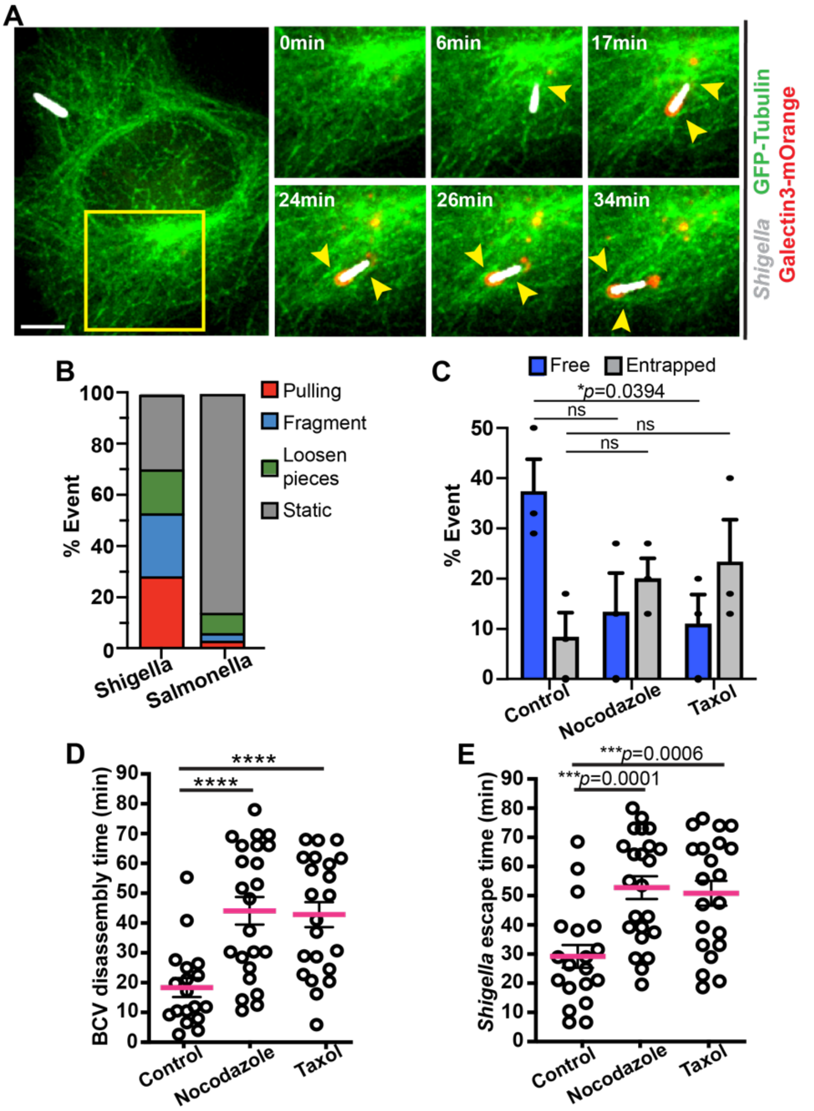

Supplementary figure 1. Microtubules are involved in the translocation of membrane remnants of the *Shigella* BCV. a Time-lapse microscopic images of localization of microtubules at entry foci of *Shigella*. Microtubules polymerized at the entry foci prior to initial BCV damage. Images were captured every 30s and maximum z-projection of stacks of the representative infection focus is shown. Yellow arrowheads indicate the localization of newly formed microtubules around the invading bacterium and the perforated BCV. Scale bar is 5 µm. **b** Quantification of BCV disassembly status during *Shigella* or *Salmonella* infection as observed in Figure 1**c**. n = two independent replicates in each condition. **c** Analysis of the fates of individual *Shigella* BCVs in cells treated with Nocodazole and Taxol. Cells treated with DMSO served as control. The bars represent mean±SEM of three independent replicates in each condition. Two-tailed Welch's t-tests were performed (ns: non-significant; *p=0.0394). Analysis of the **d** time of *Shigella* BCV disassembly and **e** *Shigella* escape time in cells. n>20 infected cells in three independent replicates in each condition. The bars (magenta) represent the mean±SEM. Statistical analysis used two-tailed Welch's t-test, with reported p-values for significance comparison (****p<0.0001).

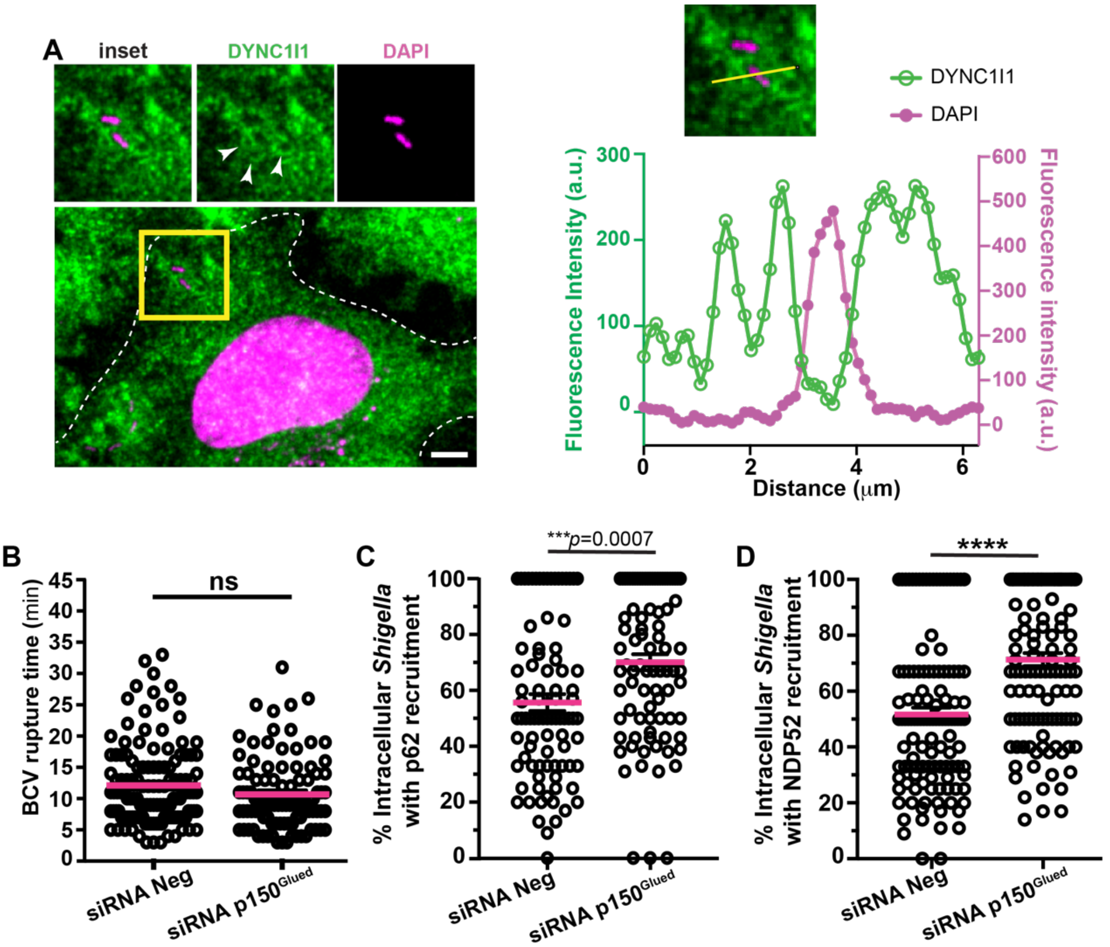

Supplementary figure 2. Dynein is recruited on *Shigella* IAMs. a Confocal images of representative infection focus of *Shigella*-infected HeLa. Inset of infection focus is highlighted in yellow, where *Shigella* and nuclei were stained by DAPI (magenta). A line (yellow) across the intracellular *Shigella* was drawn and the fluorescence intensity of DYNC1I1 and DAPI across the line (from left to right) was plotted. (right panel). Immunofluorescence staining of dynein (DYNC1I1) (green) indicated that dynein was localized on the IAMs at very close proximity to the bacteria (indicated by white arrowheads). Scale bar is 5 μm. **b** Time-lapse microscopic analysis of the time of BCV rupture during *Shigella* infection of cells subjected to RNA interference of non-targeting control (siRNA Neg) versus $p150^{Glued}$ subunit depletion (siRNA $p150^{Glued}$). n>95 infected cells in three independent replicates. The bar (magenta) represents the mean. Analysis of the recruitment of **c** p62 and **d** NDP52 to intracellular *Shigella* at 45 min-post infection in cells subjected to RNA interference of non-targeting control (siRNA Neg) versus depletion of the subset of dynein activating adaptors. n>85 infected cells in three independent replicates in each condition. The bars (magenta) represent the mean. In this figure, statistical analysis used two-tailed Welch's t-test, with reported *p*-values for significance comparison (ns: non-significant; ***p<0.001; ****p<0.0001).

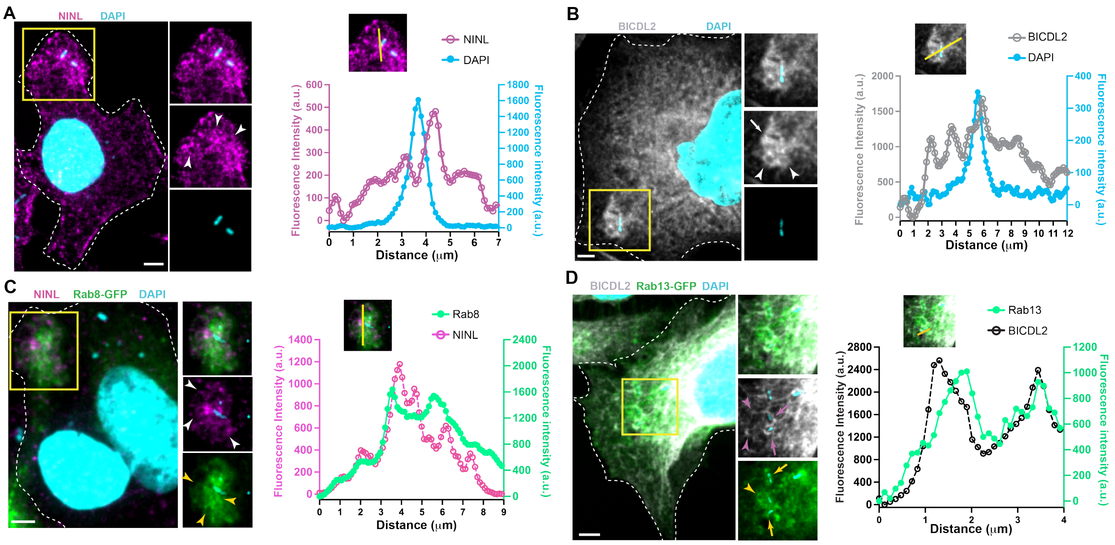

Supplementary figure 3. Rab8 and Rab13 are recruited to *Shigella* invasion sites, involving dynein activating adapter NINL and BICDL2. Confocal images of z-projections of representative infection foci of *Shigella*-infected HeLa. Insets of infection foci are highlighted in yellow, where *Shigella* and nuclei were stained by DAPI (cyan). Localization of **a** FLAG-tagged NINL (magenta) and **b** BICDL2 (grey) were studied by immunofluorescence. A line (yellow) across the intracellular *Shigella* was drawn and the fluorescence intensity of NINL and BICDL2 relative to DAPI across the line (from left to right) was plotted. (right panels). Dynein activating adaptors were present on the infection-associated macropinosomes (indicated by white arrowheads). BICDL2 was also found to be localized to very close proximity to the bacteria (as indicated by the white arrows). Scale bars are 5 μm. Localization of **c** FLAG-tagged NINL (magenta) and Rab8-GFP (green) and **d** BICDL2 (grey) and Rab13-GFP (green) were studied by immunofluorescence. Plots of fluorescence intensity of NINL and BICDL2 relative to their interacting Rab GTPases across the yellow line (from top to bottom or from left to right, respectively) showed that NINL and BICDL2 were localized to their interacting Rab GTPases at *Shigella* infection foci. (right panels). It was found that NINL (as indicated by white arrowheads) colocalized on some Rab8A-positive IAMs (as indicated by yellow arrowheads) and BICDL2 (indicated by pink arrowheads) colocalized on Rab13-positive IAMs (indicated by yellow arrowheads). BICDL2 and Rab13 was also found to be localized to very close proximity to the bacteria (as indicated by the pink and yellow arrows, respectively). Scale bars are 5 μm.

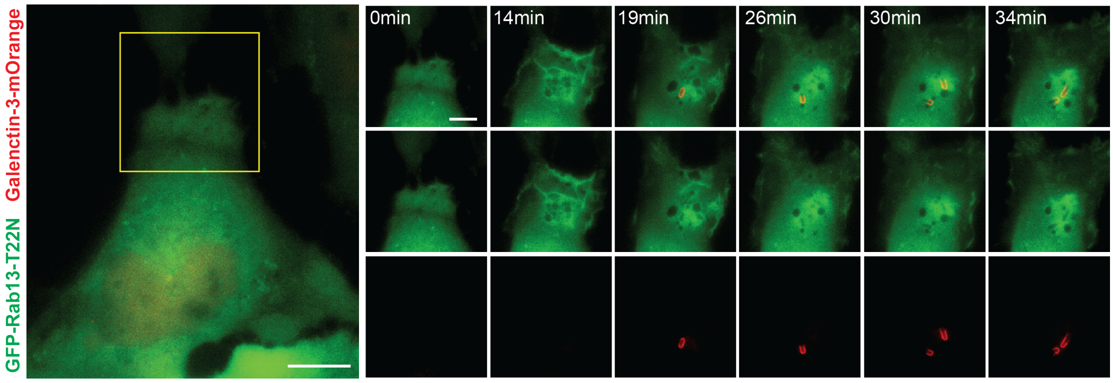

Supplementary figure 4. Rab13-T22N no longer localized on *Shigella* BCV and IAMs. Time-lapse microscopic images of the recruitment of Rab13-T22N (green) in transfected HeLa cells. Galectin-3-mOrange (red) was used as a marker of vacuolar rupture. Images were captured every minute and the z-projections of representative infection focus are shown. Scale bars are 10 μm.

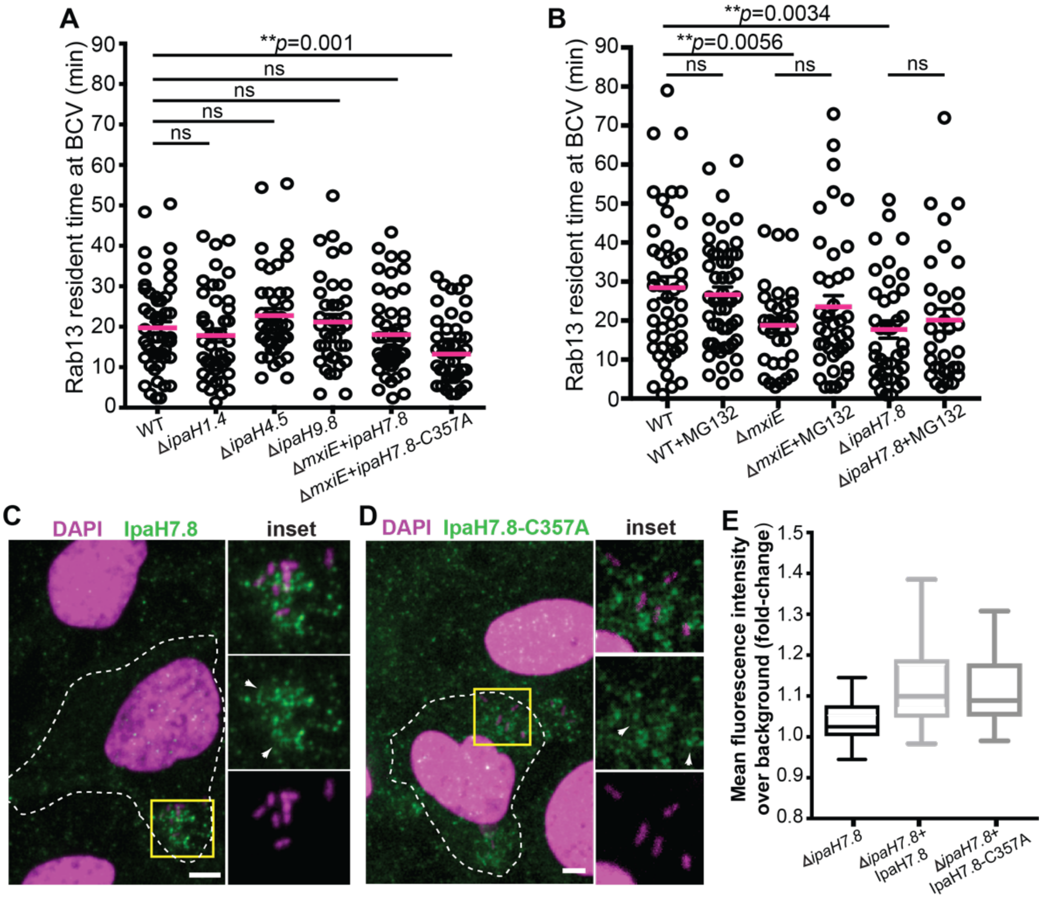

Supplementary figure 5. IpaH7.8 mediates the recruitment and retention of Rab13 on the membrane remnants of *Shigella* BCV. Time of Rab13 residence at *Shigella* BCV **a** in the presence of wild-type and different *Shigella* mutants and **b** in the presence of wild-type, *mxiE* mutant and *ipaH7.8* mutant with or without MG132 treatment. n>30 infected cells in three independent replicates. The bars (magenta) represent the mean. Confocal images of z-projection of representative infection foci of HeLa infected with *ipaH7.8* mutant complemented with FLAG-tagged wild-type IpaH7.8 or *ipaH7.8* mutant complemented with FLAG-tagged IpaH7.8-C357A mutant. Insets of infection foci were highlighted in yellow, where *Shigella* and nuclei were stained by DAPI (magenta). Immunofluorescence staining of FLAG-tagged **c** IpaH7.8 (green) and **d** IpaH7.8-C357A (green) indicated that IpaH7.8 variants were recruited to infection foci and localized in very close proximity to the bacteria (indicated by white arrowheads). Scale bars are 5 µm. **e** Mean fluorescence intensity (in terms of fold-change) relative to the background fluorescence of FLAG-tagged IpaH7.8 variants around intracellular *Shigella* at the infection foci as observed in **c-d**. n>35 infected cells in three independent replicates. In this figure, statistical analysis used two-tailed Welch's t-test, with reported *p*-values for significance comparison (ns: non-significant; $**p<0.01$).

S1 Table. Some subunits of dynein motor, kinesins and motor-regulating Rab GTPases are enriched at the IAMs (INF-M *vs* INF-NM). Statistical analysis used a paired t-test with the R package limma (*Nucleic Acids Res.* 43, e47 (2015) and adaptive Benjamini-Hochberg correction of the *p* values were performed using the R package cp4p (*Proteomics*, 16, 29-32 (2016)

			Label-free Quantification (LFQ) intensity							
**Protein**	**$\log_2$ Fold-change**	**Adjusted p-value**	**INF-M-1**	**INF-M-2**	**INF-M-3**	**INF-NM-1**	**INF-NM-2**	**INF-NM-3**	**# Peptides**	**Molecular Weight (kDa)**
DYNC1H1	1.42	2.99E-04	8.7E+09	7.0E+09	3.2E+09	2.2E+09	2.5E+09	1.9E+09	233	532.4
DYNC1LI1	1.34	6.57E-04	3.1E+08	2.3E+08	7.5E+07	7.8E+07	9.3E+07	4.5E+07	19	56.578
DYNC1LI2	1.17	6.07E-04	1.0E+08	8.5E+07	3.1E+07	2.8E+07	3.2E+07	2.7E+07	11	45.004
KIF1B	NA	NA	1.8E+07	1.8E+07	0	0	0	0	13	130.36
KIF4A	NA	NA	2.8E+08	1.0E+04	1.7E+08	0	0	0	28	139.88
KIFC1	NA	NA	1.8E+07	1.4E+07	0	0	0	0	6	73.747
KIF11	1.61	1.71E-04	9.1E+07	6.3E+07	5.3E+07	2.7E+07	2.6E+07	1.6E+07	21	119.16
KIF14	4.72	7.02E-06	7.5E+08	4.3E+08	4.6E+08	1.4E+07	1.9E+07	2.9E+07	59	184.49
KIF20A	NA	NA	1.1E+08	8.9E+07	6.3E+07	0	0	0	22	100.28
KIF20B	NA	NA	0	1.7E+09	4.3E+09	0	0	0	2	199.81
KIF23	4.78	1.94E-06	2.5E+09	3.6E+09	2.4E+09	0	9.3E+07	1.1E+08	52	110.06
KIF2A	1.08	2.99E-04	5.7E+07	6.2E+07	5.2E+07	2.4E+07	2.6E+07	3.1E+07	10	76.954
Rab8A	1.11	4.91E-04	2.0E+08	9.9E+07	8.3E+07	7.2E+07	4.4E+07	5.1E+07	8	23.668
Rab13	3.83	6.78E-03	1.9E+08	9.3E+07	5.4E+07	4.1E+07	4.6E+07	0	10	22.774
