# Supplementary material for: Microtubules provide force to promote membrane uncoating in vacuolar escape for a cyto-invasive bacterial pathogen
Source: Nat Commun. 2024 Feb 5;15:1065. doi: 10.1038/s41467-024-45182-6 (PMC10844605; doi:10.1038/s41467-024-45182-6)
Supplement: Supplementary file 10 — Reporting Summary [file 41467_2024_45182_MOESM10_ESM.pdf]

nature portfolio

Corresponding author(s): Jost Enninga, John Rohde

Last updated by author(s): Dec 4, 2023

Reporting Summary

Nature Portfolio wishes to improve the reproducibility of the work that we publish. This form provides structure for consistency and transparency in reporting. For further information on Nature Portfolio policies, see our Editorial Policies and the Editorial Policy Checklist.

Statistics

For all statistical analyses, confirm that the following items are present in the figure legend, table legend, main text, or Methods section.

n/a	Confirmed	
☐	☒	The exact sample size (*n*) for each experimental group/condition, given as a discrete number and unit of measurement
☐	☒	A statement on whether measurements were taken from distinct samples or whether the same sample was measured repeatedly
☐	☒	The statistical test(s) used AND whether they are one- or two-sided<br>*Only common tests should be described solely by name; describe more complex techniques in the Methods section.*
☒	☐	A description of all covariates tested
☒	☐	A description of any assumptions or corrections, such as tests of normality and adjustment for multiple comparisons
☐	☒	A full description of the statistical parameters including central tendency (e.g. means) or other basic estimates (e.g. regression coefficient) AND variation (e.g. standard deviation) or associated estimates of uncertainty (e.g. confidence intervals)
☐	☒	For null hypothesis testing, the test statistic (e.g. *F*, *t*, *r*) with confidence intervals, effect sizes, degrees of freedom and *P* value noted<br>*Give P values as exact values whenever suitable.*
☒	☐	For Bayesian analysis, information on the choice of priors and Markov chain Monte Carlo settings
☒	☐	For hierarchical and complex designs, identification of the appropriate level for tests and full reporting of outcomes
☒	☐	Estimates of effect sizes (e.g. Cohen's *d*, Pearson's *r*), indicating how they were calculated

Our web collection on statistics for biologists contains articles on many of the points above.

Software and code

Policy information about availability of computer code

Data collection	Live imaging was performed on DeltaVision Elite (GE Healthcare). Fixed imaging was performed on Perkin Elmer Ultraview spinning disk confocal microscope using software Volocity.
Data analysis	Images were processed using the built-in deconvolution analysis module on DeltaVision Elite (GE Healthcare) or FIJI version 2.1.0/ 1.53c was used for image analysis (available for download from https://fiji.sc); Statistical analysis was performed using software GraphPad Prism version 8.0 or 9.3.1.

For manuscripts utilizing custom algorithms or software that are central to the research but not yet described in published literature, software must be made available to editors and reviewers. We strongly encourage code deposition in a community repository (e.g. GitHub). See the Nature Portfolio guidelines for submitting code & software for further information.

Data

Policy information about availability of data

All manuscripts must include a data availability statement. This statement should provide the following information, where applicable:

- Accession codes, unique identifiers, or web links for publicly available datasets
- A description of any restrictions on data availability
- For clinical datasets or third party data, please ensure that the statement adheres to our policy

The data that support the findings of this study are available within the article and supplementary information. Source data are provided with this paper.

Research involving human participants, their data, or biological material

Policy information about studies with human participants or human data. See also policy information about sex, gender (identity/presentation), and sexual orientation and race, ethnicity and racism.

Reporting on sex and gender	N/A
Reporting on race, ethnicity, or other socially relevant groupings	N/A
Population characteristics	N/A
Recruitment	N/A
Ethics oversight	N/A

Note that full information on the approval of the study protocol must also be provided in the manuscript.

Field-specific reporting

Please select the one below that is the best fit for your research. If you are not sure, read the appropriate sections before making your selection.

☒ Life sciences ☐ Behavioural & social sciences ☐ Ecological, evolutionary & environmental sciences

For a reference copy of the document with all sections, see nature.com/documents/nr-reporting-summary-flat.pdf

Life sciences study design

All studies must disclose on these points even when the disclosure is negative.

Sample size	No statistical methods were used to predetermine the sample size. All the analyses were from at least three independent experimental data sets. All sample sizes have are indicated in the figure legends.
Data exclusions	No data were excluded from the analysis.
Replication	All the analyses were performed in triplicate with similar and consistent results.
Randomization	All infection assays (with and without the use of siRNA; expressing wild-type or the mutant proteins or in the presence of wild-type or gene knock-out or the gene complementation strains) were performed under the same conditions. Data were collected and processed in parallel. Therefore, no randomization was necessary in our experiments and was not performed.
Blinding	Microscopic data were acquired and quantitative analyses were performed blindly until interpretation of the results.

Reporting for specific materials, systems and methods

We require information from authors about some types of materials, experimental systems and methods used in many studies. Here, indicate whether each material, system or method listed is relevant to your study. If you are not sure if a list item applies to your research, read the appropriate section before selecting a response.

Materials & experimental systems

n/a	Involved in the study	
☐	☒	Antibodies
☐	☒	Eukaryotic cell lines
☒	☐	Palaeontology and archaeology
☒	☐	Animals and other organisms
☒	☐	Clinical data
☒	☐	Dual use research of concern
☒	☐	Plants

Methods

n/a	Involved in the study	
☒	☐	ChIP-seq
☒	☐	Flow cytometry
☒	☐	MRI-based neuroimaging

Antibodies

Antibodies used	Rabbit anti-LC3 (Abcam, Cat#ab48394; RRID: AB_881433); Mouse anti-DYNC1I1 (Sigma Aldrich, Cat#MAB1618; RRID: AB_2246059); Mouse anti-FLAG (M2) (Sigma Aldrich, Cat#F3165; RRID: AB_259529); Rabbit anti-BICDL2 (Thermo Fisher, Cat#PA5-60293; RRID:

	AB_2639475); Rabbit anti-NDP52 (Abcam, Cat#ab68588; RRID: AB_1640255); Rabbit anti-p62 (MBLbio, Cat#PM045; RRID: AB_1279301); Rabbit anti-Shigella (Abcam, Cat#ab65282; RRID: AB_1142846); Goat anti-rabbit IgG (H+L) highly cross-adsorbed secondary antibody, Alexa Fluor488 (Thermo Fisher, Cat#A11034; RRID: AB_2576217); Goat anti-rabbit IgG (H+L) cross-adsorbed secondary antibody, Cy3 (Thermo Fisher, Cat#A10520; RRID: AB_10563288); Goat anti-mouse IgG (H+L) cross-adsorbed secondary antibody, FITC (Thermo Fisher, Cat#F-2761; RRID: AB_2536524); Goat anti-mouse IgG (H+L) cross-adsorbed secondary antibody, Cy3 (Thermo Fisher, Cat#A10521; RRID: AB_10373848)
Validation	Rabbit anti-LC3 (https://scicrunch.org/resolver/RRID:AB_881433?q=&i=61709c722e82a7124696086d); Mouse anti-DYNC1I1 (https://scicrunch.org/resolver/AB_2246059); Mouse anti-FLAG-M2 (https://scicrunch.org/resolver/AB_259529); Rabbit anti-BICDL2 (Immunogen sequence: EEKEVEVAKL QDEISLQQAE LQSLREELQR QKELRAQEDP GEALHSALSD RDEAVNKALE LSLQLNRVSL ERDSLSRELL - React to Human; Highest antigen sequence identity to the following orthologs: Mouse - 80%, Rat - 83%); Rabbit anti-NDP52 (https://www.abcam.com/products/primary-antibodies/ndp52-antibody-ab68588.html); Rabbit anti-p62 (https://www.mblbio.com/bio/g/dtl/A/?pcd=PM045); Rabbit anti-Shigella (https://www.abcam.com/products/primary-antibodies/shigella-antibody-ab65282.html)

Eukaryotic cell lines

Policy information about cell lines and Sex and Gender in Research

Cell line source(s)	Human epithelial HeLa cells (ATCC, cat. no. ATCC® CCL-2; RRID: CVCL_0030); Human colon adenocarcinoma Caco-2/TC-7 (kind sharing from Philippe Sansonetti lab)
Authentication	HeLa cells were sent to ATCC Cell Line Authentication Service using Short Tandem Repeat analysis as described in ANSI Standard (ASN-0002). Caco-2/TC-7 cells were not authenticated
Mycoplasma contamination	All cell lines were tested negative for mycoplasma.
Commonly misidentified lines (See ICLAC register)	The cell lines used in the study are not in the commonly misidentified lines list.
