# Supplementary material for: Microtubules provide force to promote membrane uncoating in vacuolar escape for a cyto-invasive bacterial pathogen
Source: Nat Commun. 2024 Feb 5;15:1065. doi: 10.1038/s41467-024-45182-6 (PMC10844605; doi:10.1038/s41467-024-45182-6)
Supplement: Supplementary file 2 — Peer Review File [file 41467_2024_45182_MOESM2_ESM.pdf]

nature portfolio

Peer Review File

Microtubules provide force to promote membrane uncoating in vacuolar escape for a cyto-invasive bacterial pathogen

Open Access This file is licensed under a Creative Commons Attribution 4.0 International License, which permits use, sharing, adaptation, distribution and reproduction in any medium or format, as long as you give appropriate credit to the original author(s) and the source, provide a link to the Creative Commons license, and indicate if changes were made. In the cases where the authors are anonymous, such as is the case for the reports of anonymous peer reviewers, author attribution should be to 'Anonymous Referee' followed by a clear attribution to the source work. The images or other third party material in this file are included in the article's Creative Commons license, unless indicated otherwise in a credit line to the material. If material is not included in the article's Creative Commons license and your intended use is not permitted by statutory regulation or exceeds the permitted use, you will need to obtain permission directly from the copyright holder. To view a copy of this license, visit http://creativecommons.org/licenses/by/4.0/.

REVIEWER COMMENTS

Reviewer #1 (Remarks to the Author):

With a focus on cutting edge microscopy and cell biology approaches, the authors investigate subversion of microtubule transport machinery during Shigella infection and conclude that mechanical forces coordinate the efficient escape of Shigella from the phagocytic vacuole to the cytosol.

The role of microtubules in the case of cytosolic Shigella infection is relatively poorly understood (as compared to actin). The cell biology and use of innovative Shigella mutants is performed at a high level. Although the precise role of all molecules under investigation are not fully shown, this is likely difficult to elucidate using microscopy alone. The majority of readouts are related to BCV disassembly / escape. Can other experimental approaches be used to support the overall impact of the new infection biology discovered here? (for this manuscript or in the case of future work)

That microtubules provide force to promote membrane uncoating in vacuolar escape is specific to Shigella (not Salmonella). Are microtubules / multidirectional forces exerted for membrane uncoating only important for phagosome escape? Can the same principles apply to bacteria trying to escape from the autophagosome? Additional experiments are not relevant here, but can authors speculate on the generalisability of their findings.

LC3 recruitment is measured in Figure 3, to highlight defects in autophagy escape. What is the precise role of LC3 recruitment / autophagy here? To repair damaged phagosomal membrane or to target bacteria to destruction?

Reviewer #2 (Remarks to the Author):

The study of Chang and colleagues proposes that microtubule-dependent transport contributes to disruption of the pathogen-containing vacuoles (PCV) and the removal of the remnant membranes of disrupted vacuoles. The main infection model is infection of epithelial cells by Shigella flexneri, and use of the previously established host cell sensor Galectin-3 to detect damaged PCV, and to follow the cellular fate of membranes originating from damaged PCV. The work reports that microtubule (MT)-dependent transport is required to move PCV membranes, a role of MT motor protein dynein, host cell Rab13 is required to initiate membrane uncoating, and Shigella T3SS effector protein IpaH7.8 is involved in recruitment of Rab13 to PCV.

The contribution of the host cell to release of an intracellular pathogen from the PCV is an interesting new observation. This may help to further understand the manipulation of generic host cell mechanism by pathogens. The observation that host cell membranes are transported by MT-dependent transport is per se not novel, but the pathogenic manipulation could reveal an new paradigm. In this sense, a number of technical and conceptual issue need to be addressed to bring this observation to understanding of physiological consequences and mechanisms.

Specific comments:

My main criticism if the rather indirect conclusion about the existence of a PCV, the presence of intracellular bacteria, and the damage of the PCV. The study entire relies on recruitment of Galectin3-mOrange (Gal3) to sites of PCV damage, and for the later fate of membranes decorated with Gal3. Although the Gal3 sensor has been established in previous work of the authors as host cell factor recruited to sites of damage of PCV, I consider it as essential to include labeling of bacteria and, if possible, a marker for the non-damaged PCV. It is at all not intuitive to report on intracellular bacteria and their PCV if neither is visualized. Given the excellent expertise of the authors in volumetric electron microscopy, the study would also benefit from ultrastructural demonstration of PCV remnants associated with MT.

As it stands, the analyses report the MT-dependent distribution of Gal3 complexes, and conclusions on the status of the PCV and disruptive effects of the intracellular bacteria are rather indirect. If some effects of intracellular Shigella induce Gal3 aggregation, this may induce subsequent disposal be MT-dependent transport. Given the rather long intervals between frames, recordings at least one additional fluorescence signal should be feasible.

The sole reliance on Gal3 also limits the interpretation of mechanisms of PCV disruption. Since in the current setup Gal3 has been recruited to PCV and prior damage is required for recruitment, any subset event acts on damaged PCV. Whether MT contribution to uncoating is important successful intracellular pathogenesis is rather difficult to address. Alternative labeling and imaging strategies should be considered.

Is MT-dependent uncoating a generic mechanism of disposal of damages vacuolar compartments, or specifically induced by Shigella? The authors could extend their experimental system to controlled induction of vacuolar damage independent from Shigella action, and analyses of subsequent events.

Is MT-dependent uncoating required for the successful intracellular lifestyle of Shigella? So far, the study does not provide direct evidence for contributions of this observation to virulence. Assays to test a correlation are probably challenging, but required to set the reported observations into a context of infection biology. One may investigate if defective or inhibited uncoating affects intracellular proliferation and intercellular spread of Shigella.

Minor comments:

Please specify for the imaging data if single planes of stacks or maximum intensity projections are shown. This information is missing.

Fig. 3: please provide Suppl. Data on the experimental system. How was scoring done and which phenotypes are considered positive/negative for scoring?

Fig. S1B: Please provide statistical analyses.

Fig. S2, S3: In view of the provided examples, it is difficult to conclude on recruit of dynein or Rab8 or Rab13 to the PCV. The images provided hardly support these statements. Please include line scans to test potential alterations in distribution of these proteins, and quantitative analyses.

Responses to the Reviewers

Color code: The reviewers' comments are in black and our responses are in blue.

Reviewer #1 (Remarks to the Author):
With a focus on cutting edge microscopy and cell biology approaches, the authors investigate subversion of microtubule transport machinery during Shigella infection and conclude that mechanical forces coordinate the efficient escape of Shigella from the phagocytic vacuole to the cytosol.

The role of microtubules in the case of cytosolic Shigella infection is relatively poorly understood (as compared to actin). The cell biology and use of innovative Shigella mutants is performed at a high level. Although the precise role of all molecules under investigation are not fully shown, this is likely difficult to elucidate using microscopy alone. The majority of readouts are related to BCV disassembly / escape. Can other experimental approaches be used to support the overall impact of the new infection biology discovered here? (for this manuscript or in the case of future work)

We thank the reviewer for the suggestions. We have performed additional experiments (e. g. plaque infection assay, autophagic makers recruitment assay, etc.) to support our findings. These experiments show that intracellular proliferation and cell-to-cell spread of *Shigella* is impaired when the microtubule network is disrupted by microtubule-depolymerization nocodazole. Our results also demonstrate that *Shigella* is being targeted to canonical autophagic pathway (as shown by the increase in recruitment of autophagic markers, p62 and NDP52) at defective BCV membrane uncoating. These additional findings collectively demonstrate the crucial roles of the microtubule network and the associated dynein motor for the dissimilation of *Shigella* and possibly other related cytosolic bacterial pathogens. The results of these assays have been included in the revised manuscript and will be further discussed in this letter.

That microtubules provide force to promote membrane uncoating in vacuolar escape is specific to Shigella (not Salmonella). Are microtubules / multidirectional forces exerted for membrane uncoating only important for phagosome escape? Can the same principles apply to bacteria trying to escape from the autophagosome? Additional experiments are not relevant here, but can authors speculate on the generalisability of their findings.

For the membranous compartments (e. g. phagosomes) to be uncoated on the microtubule network, the presence of all the necessary molecular players, including the microtubules and the microtubule-based molecular motors that would exert disproportionated forces on the uncoating membranes, in the microenvironment are of utmost importance.

In the case of uncoating of *Shigella*-containing phagosomes, we demonstrated in this submitted manuscript that microtubules and the microtubule-based molecular motor dynein are present on the BCV remnants. Apart from that, we found that components of

the dynein motor and several kinesins were considerably enriched on the infection-associated macropinosomes (IAMs) in our previously published proteomic analysis on the composition of IAMs (SI table 1; *PLoS Pathog.,* 2020, 16, e1008822). Our lab is currently investigating the precise involvement of these molecular motors on facilitating the fragmentation of the perforated *Shigella* BCV and the BCV membrane uncoating. Based on our findings reported in this submitted manuscript, we confirmed that microtubules and dynein (whose recruitment mediated by IpaH7.8-Rab13 interaction) are localized on BCV membrane remnants. Therefore, this work on the hijack of microtubule network and its associated molecular motors by *Shigella* signifies a unique paradigm in the establishment of intracellular niche by bacterial pathogens.

Notwithstanding, the observation on the involvement of microtubule-based motor (kinesin) for the uncoating of the viral capsid of adenovirus (*Cell Host Microbe*, **2011**, 10, 3, 210-223) is intriguingly similar to the disassembly of *Shigella* BCV. It is thus tempting to speculate that our findings are relevant to the contribution of microtubule/microtubule-based motors to the uncoating of membranous compartments or coats during pathogen invasion.

LC3 recruitment is measured in Figure 3, to highlight defects in autophagy escape. What is the precise role of LC3 recruitment / autophagy here? To repair damaged phagosomal membrane or to target bacteria to destruction?

We thank the reviewer for the feedback. We have set up an immunofluorescence-based assay to study the localization of other autophagic markers (p62, NPD52) in WT and $p150^{Glued}$-KO cells. Our data showed that the probability of recruitment of the early autophagic marker LC3 (Figure 2E) as well as the late autophagic markers p62 (Figure S2C) and NDP52 (Figure S2D) to the vicinity of the intracellular *Shigella* increased if BCV uncoating is impaired. The larger impact of LC3 recruitment during *Shigella* infection is not fully resolved. We favor the notion that LC3 recruitment may increase the chances of targeting the bacteria to destruction via the canonical autophagic pathway in this case.

Reviewer #2 (Remarks to the Author):

The study of Chang and colleagues proposes that microtubule-dependent transport contributes to disruption of the pathogen-containing vacuoles (PCV) and the removal of the remnant membranes of disrupted vacuoles. The main infection model is infection of epithelial cells by Shigella flexneri, and use of the previously established host cell sensor Galectin-3 to detect damaged PCV, and to follow the cellular fate of membranes originating from damaged PCV. The work reports that microtubule (MT)-dependent transport is required to move PCV membranes, a role of MT motor protein dynein, host cell Rab13 is required to initiate membrane uncoating, and Shigella T3SS effector protein IpaH7.8 is involved in recruitment of Rab13 to PCV.

The contribution of the host cell to release of an intracellular pathogen from the PCV is an interesting new observation. This may help to further understand the manipulation of generic host cell mechanism by pathogens. The observation that host cell membranes are transported by MT-dependent transport is per se not novel, but the pathogenic manipulation could reveal an new paradigm. In this sense, a number of technical and conceptual issue need to be addressed to bring this observation to understanding of physiological consequences and mechanisms.

Specific comments:
My main criticism if the rather indirect conclusion about the existence of a PCV, the presence of intracellular bacteria, and the damage of the PCV. The study entire relies on recruitment of Galectin3-mOrange (Gal3) to sites of PCV damage, and for the later fate of membranes decorated with Gal3. Although the Gal3 sensor has been established in previous work of the authors as host cell factor recruited to sites of damage of PCV, I consider it as essential to include labeling of bacteria and, if possible, a marker for the non-damaged PCV. It is at all not intuitive to report on intracellular bacteria and their PCV if neither is visualized.

We thank the suggestions of the reviewer. We performed additional experiments to include the use of fluorescently-labeled *Shigella*, together with galectin-3, to monitor the localization of microtubules upon *Shigella* host cell entry in live imaging. The results will be discussed in this letter below.

Furthermore, studies from our team and other teams in the *Shigella* community show that *Shigella* is a professional cytosolic bacterial pathogen that efficiently ruptures its vacuole upon host cell entry (*Cell Microbiol*., 2010, 12(4):545-56; *PLoS Pathog*. 2016, 12(5):e1005602; *PNAS*, 2016, 113(17):4794-9). There is still very little knowledge available about the composition of the *Shigella*-containing vacuole. Therefore, it has remained technically challenging to use lipid probes or protein-based markers to specifically label the vacuole.

Given the excellent expertise of the authors in volumetric electron microscopy, the study would also benefit from ultrastructural demonstration of PCV remnants associated with MT.

Microtubules are unfortunately not clearly visible using large volume EM approaches, which only provide insights on the 3D organization of endomembrane compartments. Microtubules are visible by cryo tomography. However, including data from such experimentation is beyond the scope of this manuscript and would represent a manuscript in itself due to the overall complexity of the workflows and analysis.

As it stands, the analyses report the MT-dependent distribution of Gal3 complexes, and conclusions on the status of the PCV and disruptive effects of the intracellular bacteria are rather indirect. If some effects of intracellular Shigella induce Gal3 aggregation, this may induce subsequent disposal be MT-dependent transport. Given the rather long intervals between frames, recordings at least one additional fluorescence signal should be feasible.

We thank the suggestions from the reviewer. We performed additional experiment using fluorescently-labeled bacteria, together with galectin-3, to monitor the localization of microtubules and the BCV disassembly in live imaging. As shown in Figure S1A, our data demonstrated that polymerization of microtubules occurred *in situ* at the *Shigella* entry focus prior to BCV rupture. After that, intense galectin-3 signals only appeared surrounding the invading *Shigella*, which marked the damaged BCV. In this particular example, the membrane remnant was pulled from the opposing end of the moving bacterium on a microtubule track.

We elucidated in this submitted manuscript that the presence of critical host factors (microtubule network, specific Rab GTPase and molecular motors) and bacterial factor (bacterial effector IpaH7.8) in vicinity determines the efficiency of *Shigella* BCV uncoating. In this submitted manuscript, we have included *Salmonella typhimurium,* which also initiates the vacuolar damage within the first hour post-infection as a parallel analysis. We demonstrated that the damaged *Salmonella*-containing vacuole (as marked by the galectin-3 signal) did not disassemble in our tested condition. This observation implies that galectin-3 labeling on membranes of damaged bacterial vacuoles does not lead to microtubule-dependent transport. We also verified using *Shigella* bacterial mutants that the movement of the membrane remnants on the microtubules was impaired and galectin-3 labeled BCV failed to be efficiently uncoated, implicating that the process of BCV disassembly and uncoating is attributable to the hijack of microtubule-based transport by the invading *Shigella*.

The sole reliance on Gal3 also limits the interpretation of mechanisms of PCV disruption. Since in the current setup Gal3 has been recruited to PCV and prior damage is required for recruitment, any subset event acts on damaged PCV. Whether MT contribution to uncoating is important successful intracellular pathogenesis is rather difficult to address. Alternative labeling and imaging strategies should be considered.

Considering that *Shigella* is a professional cytosolic bacterium, all the BCVs are ruptured in our tested conditions. Thus, the focus of this work is to investigate the fate of all the damaged vacuoles after their initial damage and how *Shigella* evade immune signaling elicited from the damaged BCV membrane. It should be noted that BCV lysis is independent of the microtubule network as BCV lysis occurs even the microtubules are disrupted in the presence of the microtubule-depolymerizing drug nocodazole or microtubule-stabilizing drug taxol.

We thank the reviewer for the feedback. To investigate if microtubule-dependent BCV uncoating is relevant to the intracellular pathogenesis of *Shigella*, we analyzed the sizes of plaques formed at 18 hr post-infection in the presence of microtubule-depolymerizing drug nocodazole. Our results showed that the sizes of plaques were significantly reduced in the presence of the drug in the wild-type and *ipaH7.8* mutant complemented with wild-type IpaH7.8 (Δ*ipaH7.8*+IpaH7.8) (Figure 5G), whereas plaque sizes of the mutants that exhibited inhibited BCV uncoating (i.e. *ipaH7.8* mutant or *ipaH7.8* mutant complementing with the catalytically inactive IpaH7.8-C357A) were obviously smaller. This result corroborates the notion that microtubule network contributes to the successful cytosolic escape and intracellular pathogenesis of *Shigella*. We have included this result and the discussion on this in the manuscript.

Is MT-dependent uncoating a generic mechanism of disposal of damages vacuolar compartments, or specifically induced by Shigella? The authors could extend their experimental system to controlled induction of vacuolar damage independent from Shigella action, and analyses of subsequent events.

We thank the suggestion from the reviewer. However, we are technically unable to induce robust and controlled vacuolar damage independent of *Shigella* action - It is in our hands only possible to induce damage of smaller vesicular compartments (and to visualize such damage), however for them we cannot use optical imaging to monitor the movements of membrane remnants due to their small size.

In order to address this issue to investigate if the microtubule network is specifically recruited and hijacked by *Shigella*, we included *Salmonella typhimurium,* which also initiates the vacuolar damage within the first hour post-infection as a parallel analysis. We found that vast majority of the damaged *Salmonella*-containing vacuoles remained static during our course of live microscopic analysis (Figure S1B). Apart from that, as shown in Figure 1D, newly formed microtubules (as marked by mEmerald-CLIP170) were not bordered to the damaged *Salmonella*-containing vacuole (as marked by galectin-3-mOrange). Our data thus demonstrated that MT-dependent uncoating is not a generic mechanism of the disposal of damaged vacuoles as uncoating of the damaged *Salmonella*-containing vacuole was not observed in our tested conditions. The vast difference in the fate of the damaged vacuoles of *Shigella* and *Salmonella* implicates that the manipulation of microtubules in the disposal of damaged membrane remnants is unique to *Shigella* infection.

Our work elucidates a similar observation on the involvement of microtubule-based motor (kinesin) for the uncoating of the viral capsid of adenovirus (*Cell Host Microbe*, **2011**, 10, 3, 210-223). We speculate that our findings may be relevant to the contribution of microtubule/microtubule-based motors to the uncoating of membranous compartments or coats during other pathogen invasion in addition to *S. flexneri*.

Is MT-dependent uncoating required for the successful intracellular lifestyle of Shigella? So far, the study does not provide direct evidence for contributions of this observation to virulence. Assays to test a correlation are probably challenging, but required to set the reported observations into a context of infection biology. One may investigate if defective or inhibited uncoating affects intracellular proliferation and intercellular spread of Shigella.

We thank the reviewer for the feedback. In order to investigate the effects of the microtubule-dependent defective BCV membrane uncoating, we analyzed the cell-to-cell spread of *Shigella* as reflected by the sizes of plaques formed after 18 hr of infection in the absence or presence of microtubule-depolymerizing drug nocodazole. Our results showed that the sizes of plaques were significantly reduced in the presence of the drug in the wild-type and *ipaH7.8* mutant complemented with wild-type IpaH7.8 (Δ*ipaH7.8*+IpaH7.8) (Figure 5G). We also observed a noticeable reduction in plaque size in strains that exhibited defective BCV uncoating (i.e. *ipaH7.8* mutant or *ipaH7.8* mutant complementing with the catalytically inactive IpaH7.8-C357A), reiterating the role of IpaH7.8 in phagosomal escape of *Shigella*, possibly via Rab13-dynein-mediated BCV uncoating as discovered in this work. This result confirmed that the microtubule network contributes to the intercellular spread of *Shigella*. We have included this result and the discussion in the manuscript.

Minor comments:

Please specify for the imaging data if single planes of stacks or maximum intensity projections are shown. This information is missing.

The images are the maximum intensity z-projections of stacks of representative infection foci. This information has been included in the figure captions where images of the time-lapse microscopic analysis are shown.

Fig. 3: please provide Suppl. Data on the experimental system. How was scoring done and which phenotypes are considered positive/negative for scoring?

siRNAs were used to silence the specified activating adaptors in HeLa cells for 72 hrs. Infection of these siRNA-treated HeLa cells was performed and we employed a previously reported quantification method (Chang *et al.*, PLoS Pathogens, 2020, 16(8): e1008822) to analyze the BCV disassembly time and the *Shigella* escape time. The BCV disassembly time was defined as the time at which the BCV completely disintegrates and is dissociated from *S. flexneri*. The *Shigella* escape time was defined as the timing of actin tail formation, which is the time interval between the appearance of the actin cage around the BCV before rupture and the appearance of the actin tail after BCV rupture.

The data in Figure 3 were presented in the descending order of the average times determined in the respective assays (left to right). Unpaired t-tests were performed comparing between siRNA negative control and the siRNA targeting the specified gene. The colors of the x-axis labels indicate the statistical significance - red: ****p<0.0001; orange: ***p<0.001; green: **p<0.01; blue: *p<0.05; black: non-significant.

Fig. S1B: Please provide statistical analyses.
We performed the unpaired t-tests of the data, comparing the control samples with the drug-treated samples, which indicates that the comparisons are statistically insignificance except for the comparison of *Shigella* moved free of BCV membrane remnant in the control and the taxol-treated condition. We have revised the corresponding figure (now Figure S1C) accordingly. We reasoned that this is due to the large variations in this assay in the presence of the drug treatment. Although being statistically insignificant, we observed a trend in the reduction in bacteria moving free of any membrane remnants (Figure S1C). This is also reflected in the quantification of the BCV assembly time (Figure S1D) and the escape time of the intracellular bacteria (Figure S1E). We have included this discussion in the main text.

Fig. S2, S3: In view of the provided examples, it is difficult to conclude on recruit of dynein or Rab8 or Rab13 to the PCV. The images provided hardly support these statements. Please include line scans to test potential alterations in distribution of these proteins, and quantitative analyses.
We thank the reviewer for the feedback. We analyzed the distribution of dynein, Rab8 and Rab13 with reference to the intracellular bacteria, based on the fluorescence intensity along the line scans. Our analyses showed that fluorescence signals of these proteins were enriched around the intracellular bacteria (Fig. S2A and S3), implicating the recruitment of these proteins to close proximity of the bacteria. The localization of dynein activating adaptors NINL and BICDL2 were also shown to localized to membrane compartments that were decorated with their interacting Rab GTPases at the infection foci (Fig. S3). We have revised Fig. S2, Fig. S3 and the main text to describe these phenotypes precisely. Apart from that, in our live microscopy experiment, we observed that the intracellular *Shigella* was sketched with Rab13, whereas the membrane remnants that moved away from the bacteria were also decorated with Rab13 (Figure 4A). This result thus supports the localization of Rab13 on *Shigella* BCV.

REVIEWERS' COMMENTS

Reviewer #1 (Remarks to the Author):

The authors have responded to reviewers' comments to more fully support conclusions.

The manuscript is well written. Novel experiments and approaches help to distinguish this novel work from previous work from this lab, highlighting how Shigella subverts microtubules and the intracellaur transport machinery for its intracellular advantage.

Reviewer #2 (Remarks to the Author):

Comments on revised version:

In the revised version, the authors' have sufficiently addressed my comments and criticism to the initial version of the manuscript. New data were added that allow detection of intracellular Shigella during the event of BCV damage. Also, the physiological role of the microtubule-dependent uncoating was supported by new experimental data showing reduced plaque formation, i.e. reduced intercellular spread of Shigella, if uncoating if manipulated.

In case additional experimental data were not provided, the authors explain convincingly why this was not done.

In the revised version, the manuscript reports an interesting new mechanism in bacterial pathogenesis. The reported observations will probably stimulate further work if the field.
